# The establishment of the species-delimits and varietal-identities of the cultivated germplasm of *Luffa acutangula* and *Luffa aegyptiaca* in Sri Lanka using morphometric, organoleptic and phylogenetic approaches

**DOI:** 10.1371/journal.pone.0215176

**Published:** 2019-04-09

**Authors:** S. A. S. M. Kumari, N. D. U. S. Nakandala, P. W. I. Nawanjana, R. M. S. K. Rathnayake, H. M. T. N. Senavirathna, R. W. K. M. Senevirathna, W. M. D. A. Wijesundara, L. T. Ranaweera, M. A. D. K. Mannanayake, C. K. Weebadde, S. D. S. S. Sooriyapathirana

**Affiliations:** 1 Regional Agriculture Research and Development Centre, Makandura, Gonawila, North Western Province, Sri Lanka; 2 Postgraduate Institute of Science, University of Peradeniya, Peradeniya, Sri Lanka; 3 Department of Molecular Biology and Biotechnology, Faculty of Science, University of Peradeniya, Peradeniya, Sri Lanka; 4 Department of Plant, Soil and Microbial Sciences, College of Agriculture and Natural Resources, Michigan State University, East Lansing, Michigan, United States of America; Institute for Biological Research, SERBIA

## Abstract

*Luffa acutangula* and *L*. *aegyptiaca* are two vegetable species commonly found in South and South East Asia. *L*. *acutangula* is widely grown; however, *L*. *aegyptiaca* is considered as an underutilized crop. The species delimits, phylogenetic positions, and the varietal identities of *L*. *acutangula* and *L*. *aegyptiaca* in Sri Lanka are not known. Thus, in the present study, we aimed to establish the species delimits and varietal identities of *L*. *acutangula* and *L*. *aegyptiaca* varieties grown in Sri Lanka using morphometric, phylogenetic and organoleptic assessments. We assessed five varieties of *L*. *acutangula* and three varieties of *L*. *aegyptiaca*. The vegetative and reproductive data were collected for the morphometric analysis and DNA sequence polymorphism of the makers *rbcL*, *trnH-psbA* and *ITS* for the phylogenetic analysis. We also conducted an organoleptic assessment based on taste parameters; aroma, bitterness, color, texture, and overall preference using the dishes prepared according to the most common Sri Lankan recipe for *Luffa*. The variation of the vegetative and reproductive traits grouped *L*. *acutangula* varieties into two distinct clusters. The *trnH-psbA* polymorphism provided the basis for the species delimits of *L*. *acutangula* and *L*. *aegyptiaca*. The *rbcL* and *ITS* polymorphisms provided the basis for the identities of the varieties of *L*. *aegyptiaca* and *L*. *acutangula* respectively. In the phylogeny, the *L*. *acutangula* varieties of Sri Lanka formed a unique clade and the *L*. *aegyptiaca* varieties formed a reciprocal monophyletic group in comparison to worldwide *L*. *aegyptiaca* reported. The taste parameters aroma, texture, color, and overall preference were significantly different among the *Luffa* varieties. The *L*. *aegyptiaca* varieties received lower preference in the organoleptic assessment. The present study sets the species delimits, phylogenetic positions and the varietal identities of the cultivated germplasm of *Luffa* and revealed the distinct morphological and organoleptic properties of each variety.

## Introduction

*Luffa* spp., (sponge gourd or dishcloth gourd [1]), are belonging to family Cucurbitaceae [2]. The genus *Luffa* comprises eight species namely *L*. *aegyptiaca*, *L*. *acutangula*, *L*. *quinquefida*, *L*. *operculata*, *L*. *saccata*, *L*. *graveolens*, *L*. *echinata*, and *L*. *astorii* [3]. Many studies report the details of morphological characterization [4, 5] and phylogenetics of *Luffa* spp. [6–9]. *L*. *acutangula* (L.) Roxb. and *L*. *aegyptiaca* Miller are the domesticated species within the genus *Luffa* [10]. *Luffa* spp. have an Asian origin and scattered in tropical regions [11, 12]. *L*. *acutangula* and *L*. *aegyptiaca* are closely related species [13]. However, their intra-specific variations remain obscured. *L*. *acutangula* and *L*. *aegyptiaca* are suggested to originate in the South East Asian region, yet, the exact place of origin is not known [3].

*L*. *acutangula* and *L*. *aegyptiaca* are annual vines bearing hermaphrodite, staminate or pistillate flowers [14, 15]. *Luffa* spp. are diploid (2n = 26) with cross-pollinated behavior [9]. The tender fruits of *L*. *acutangula* and *L*. *aegyptiaca* are popular as vegetables mostly in the South and South East Asian cuisine. The fruit size exhibits a profound variation in length, shape, and color [16, 17]. The variation of the fruit morphology and fruit flavor is mainly due to the heterozygosity that is resulting from the natural cross-pollination ability of *Luffa* spp. [18]. The consumption of *Luffa* as a vegetable also improves the appetite for food [19]. The mature and dried fruit of *Luffa* spp. has a fibrous network of cellulose that can be utilized as a bathing sponge, a biodegradable filter, a sponge to clean glassware, and kitchen appliances [20, 21]. Indigenous medical practitioners use the *Luffa* fruits to treat anemia, leucoderma, tumors, and splenic enlargement. *Luffa* fruits also possess diuretic properties [22]. ‘Luffin P1’, a phytochemical in *Luffa* seeds, possesses anti-HIV-1 activity. The anti-inflammatory effects are reported in aqueous and ethyl acetate extracts of peel and pulp of the fruits [23]. The leaves of *Luffa* is used in indigenous medicine for insect bites [22]. The dried fruit powder of *Luffa* is used to rub the swollen hemorrhoids [22]. According to Yadav et al. (2016), secondary metabolites such as alkaloids, flavonoids, glycosides, steroids, and saponins are present in *L*. *aegyptiaca* and *L*. *acutangula* [24].

Sri Lanka has a wide range of *Luffa* cultivars (Table 1) belonging to *L*. *acutangula* and *L*. *aegyptiaca* [10]. *L*. *acutangula* and *L*. *aegyptiaca* are locally known as *Darawatakolu* (*Watakolu*) and *Niyan Watakolu* respectively. *L*. *acutangula* is the most popular *Luffa* species grown in Sri Lanka. The average extent of cultivation of *L*. *acutangula* is about 3952 ha in Sri Lanka, and average yield recorded in 2016 was approximately 10.23 t/ha [25]. However, *L*. *aegyptiaca* is not cultivated extensively in commercial scale and found in isolated patches of dry and intermediate zones, and home gardens [26]. Thus, *L*. *aegyptiaca* is recognized as an underutilized vegetable crop. However, the surveys have indicated that the cultivation of the improved *Luffa* varieties is famous within the Sri Lankan farming community [27] (Table 1).

**Table 1:** The *Luffa* varieties reported to be grown in Sri Lanka.

Species	Variety	Genetics	Origin	Referene
*L*. *acuangula*	*Asiri*	Open-pollinated	Sri Lanka	[27]
	*Gannoruwa Ari* (GA)	Open-pollinated	Sri Lanka	[32]
	LA33	Open-pollinated	Sri Lanka	[33]
	*Naga-F1*	Exotic-Hybrid	Thailand	[34]
	*Nadee-F1*	Exotic-Hybrid	Thailand	
*L*. *aegyptiaca*	*Niyan Watakolu* Yellow Peel (NWYP)	Open-pollinated	Sri Lanka	Regional Agriculture Research and Development Centre, Makandura, Sri Lanka
	*Niyan Watakolu* Green Peel (NWGP)	Open-pollinated	Sri Lanka	
	LF3522	Exotic-Hybrid	Unknown	Regional Agriculture Research and Development Centre, Makandura, Sri Lanka

Majority of the farmers prefer to grow *Luffa* over most of the other vegetables due to the convenience in planting and maintenance of seeds. The accurate identification of the *Luffa* varieties at the farmers level plays a crucial role in *Luffa* cultivation. Most of the varieties have been categorized into different types and landraces by the local farmers based on the morphological variations of fruits such as the color of the peel, aroma, texture and the size [28, 29]. Previous studies have employed the vegetative traits such as the size of the leaf lamina, internode length and petiole length as the parameters to evaluate the morphological variation of cultivated *Luffa* spp. [28]. Few studies have also been conducted to identify the interspecific relationships of *Luffa* spp. using their flavonoid patterns [30]. However, the interspecific relationships vary with the employed morphological and chemotaxonomic markers and their highly diverse nature [30, 31]. Thus, morphological and chemotaxonomic markers do not provide an accurate description of *Luffa* varieties [31, 6] demanding more consistent and precise techniques to assess the genetic variability among the *Luffa* varieties.

The molecular systematic approaches with morphological characterization are the most reliable tools to ascertain the accurate intra and interspecies delimitations. Thus, in the present study, we aimed to establish intra and interspecies delimits of *L*. *acutangula* and *L*. *aegyptiaca* in Sri Lanka and detect the varietal identities within the two species using morphological characterization based on vegetative and reproductive characteristics and molecular systematics. Moreover, an organoleptic assessment was carried out to understand the consumer preference on the varieties assessed in the present study. Overall it was aimed to establish an accurate varietal description of *L*. *acutangula* and *L*. *aegyptiaca* and to facilitate the breeding of improved *Luffa* varieties.

## Materials and methods

### Plant material

Five varieties of *L*. *acutangula* and three varieties of *L*. *aegyptiaca* were assessed in the present study (Table 1). The breeder seeds of the varieties *Asiri*, *Gannoruwa Ari* (GA) and LA33 of *L*. *acutangula* were obtained from the Horticultural Crop Research and Development Institute (HORDI), Peradeniya, Sri Lanka (GPS: 7.275092, 80.602877). The seeds of two exotic hybrids; *Naga-F1* and *Nadee-F1*, of *L*. *acutangula* were obtained from a seed shop at *Rajagiriya*, Sri Lanka (GPS: 6.916278, 79.917867). The seeds of *L*. *aegyptiaca* landrace; *Niyan Watakolu* Yellow Peel (NWYP), were taken from the accession maintained at the Plant Genetic Resource Centre (PGRC), *Gannoruwa*, Sri Lanka (GPS: 7.272847, 80.602064) (PGRC Accession No.: AC#013198) and the seeds of *Niyan Watakolu* Green Peel (NWGP) were received from a farmer at *Wanathawilluwa*, Puttalam, Sri Lanka (GPS: 8.187930, 79.860081). The seeds of the *L*. *aegyptiaca* hybrid; LF3522, were obtained from a seed shop at *Thalakiriyagama*, Sri Lanka (GPS: 7.807294, 80.611014).

### Plant establishment

The plants were established in the open fields at Regional Agriculture Research and Development Centre, *Makandura* (Agro-Ecological Region IL1a of Sri Lanka: average annual rainfall of 1960 mm; maximum and minimum average temperatures 31.7 and 23.0°C respectively; Red Yellow Podsolic with Alluvial soil as a top layer) [35]. The seeds were soaked overnight in water and subjected to Cruiser Chemical Treatment recommended in the Integrated Pest Management (IPM) Package for controlling the pests of the family Cucurbitaceae. The plants/varieties were arranged according to RCBD with two replicates each having eight beds. Each bed comprised of six planting pits arranged linearly. The spacing of 1.5 m × 1.5 m was maintained within and between adjacent lines. The pits were prepared each having a volume of 0.1 m^3^ filled with cattle manure and topsoil in 1:1 ratio, followed by the addition of basal N-P-K mixture in recommended rates. The contents were mixed in the pit and allowed to settle for two days. After that, three treated seeds were planted in each pit, subsequently thinned to two well-established seedlings per pit. After two weeks of the establishment, the lateral branches were removed up to 0.6 m of the main stem from the soil level to train the plants to climb the trellis.

### Morphometric measurements

The measurements were recorded for seven vegetative parameters [leaf length (LL), leaf width (LW), petiole length (PL), internode length (INL), no. of lateral shoots (NLS), root length (RTL) and root dry weight (RDW)] describing the morphology of leaves, stems, and roots. The measurements were also taken for 22 parameters of the reproductive structures [peduncle length (PDL), first flowering node (FFN), no. of days to the first male flower (DMF), no. of days to the first female flower (NFF), male to female flower ratio (MFR), number of days to harvest the first vegetable since the flowering stage (DHV), number of days to harvest since the establishment of plants (NDH), vine length at the flowering stage (VLF) and vine girth at the flowering stage (VGF), no. of fruits (FN), fruit length (FL), fruit width (FW), fruit girth (FG), no. of ribs (RN), skin thickness (ST), flesh thickness (FT), total weight (TW), seed length (SL), seed width (SW), seed thickness (SET), hundred seed weight (HSW) and no. of seeds per pod (NP)] (S1 Table lists all the abbreviations). All the morphometric measurements were collected according to International Plant Genetic Resources (IPGR) descriptors [36]. Furthermore, photographs were also taken to illustrate the stages of fruit development of each variety.

### DNA extraction and PCR

The genomic DNA was extracted from the tender leaves using the modified cetyl trimethylammonium bromide (CTAB) protocol [37]. The PCR was carried using a Thermal Cycler (TP600: Takara, Otsu Shiga, Japan) for nine DNA barcoding markers [*trnH*-*psbA*, *rbcL*, *trnL*-*trnF* spacer, *trnS*^*GCU*^-*trnG*^*UUC*^, *atpB*-*rbcL* spacer, *atpB* gene, *matK*-*trnT* spacer, *trnL* (*tRNA*-*leu* gene) and *ITS1*-*4*] (S2 Table). The PCR mixture (15 μl) comprised 5× Go Taq Green Master Mix (7.5 μl), 10 μM forward and reverse primers (0.5 μl each) and 10 μM spermidine (3.5 μl) [38]. The PCR conditions are given in S2 Table. The PCR amplicons were size separated using 2.5% agarose gel by electrophoresis [39]. As the PCR positive controls, the DNA of two apple (Spartan and Tall cox) and two rice varieties (Bg 366 and At 307) was used.

### DNA sequencing

The DNA barcoding markers *rbcL*, *trnH-psbA* and *ITS* were chosen for sequencing considering the availability of comparison sequences for phylogenetic analysis [3] (S3 Table). The PCR products were purified using QIAquick PCR purification kit (Catalog No: 28104, Qiagen, Hilden, Germany) and cycle sequenced using the automated genetic analyzer 3500 (Catalog number: 622–0010, Applied Bio System).

### Data analysis

The quantitative data collected for the reproductive and morphological parameters were subjected to normality testing, and LS-means/pdiff mean separation under General Linear Model (GLM) procedure in the statistical package SAS 9.4 (SAS Institute, NC, Cary, USA). A dendrogram was constructed combining both vegetative and reproductive data (normalized to 0–1 range) using Complete Linkage and Euclidean Distance method in the statistical package Minitab 17 (Minitab Inc., USA). The Principal Components One and Two (PC1 and PC2) calculated from all the quantitative data were used to draw the PC biplot in Minitab.

### Phylogenetic analysis

The raw sequencing data yielded for the markers *rbcL*, *trnH-psbA*, and *ITS* were initially visualized in MEGA 7 [40] to detect the initial and end noises. The datasets were trimmed and subjected independently to a Basic Local Alignment Search Tool (BLAST) search to verify the identities of the sequences. The sequences were aligned with the data given in Filipowicz et al. (2014) [3]. For each marker, separate alignments were carried out in MEGA 7 using Clustal W algorithm [41]. The sequences were manually checked to avoid the incorporation of unwanted gaps in the alignment. The three final marker data sets were combined using Sequence Matrix software [42], and the data partition matrix was made. Since chloroplast and nuclear markers were employed in the downstream phylogenetic analyses, the phylogenetic concordance must be assessed for the combined datasets. Thus the combined alignment was subjected to partition homogeneity (ILD test) analysis [43] to check the phylogenetic congruence of three markers. The combined datasets of the Sri Lankan *Luffa* spp. were analyzed using Unweighted Pair Group Method with Arithmetic mean (UPGMA) algorithm with uncorrected pairwise distances of each sequence. The combined alignment with all the *Luffa* spp. in the world (adapted from Filipowicz et al. (2014) [3]) were uploaded to PAUP (version 4.0a) [44] to carry out the neighbor-joining (NJ) tree construction. Our approach considered all the substitutions for the tree construction, and the gaps were treated as partial deletions. Maximum Likelihood (ML) tree search was also carried out in RAxML [45] using the rapid bootstrap algorithm [46]. The analysis was run for 1000 iterations, and the DNA model was selected as GTRGAMMA [47]. The bootstrap replicates were included into one tree topology using bipartition option in RAxML.

Moreover, a Bayesian analysis was carried out in MrBays [48]. Since the Bayesian tree construction associates with DNA substitution model, the model selection was carried out in Akaike Information Criteria (AIC) [49] and corrected Akaike Information Criteria (AICc) [50] in J model test [51]. Then the best nucleotide parameters were appointed in the Bayesian tree search and ran four Markov Chain Monte Carlo (MCMC) chains for fifty million cycles to probe heuristically in tree space. The trees were probed after every 5000 chain-runs, and the initial 10% of trees probed were discarded as burn-in to achieve the sampling from the independent and sufficient sample size. The analysis was set to draw a 50% majority rule consensus tree as the final output. The Maximum Likelihood, Bayesian tree searches, and model selection were carried out in CIPRES science gateway [52]. The final tree output was further modified in Figtree version 1.4.3 [53].

### Evaluation of consumer preference

*L*. *acutangula* and *L*. *aegyptiaca* fruit samples from each variety were cooked according to the most common recipe for *Luffa* dishes in Sri Lanka. One kilogram of fresh fruit sample from each variety was separately cooked for five mins in slow heat by adding 50ml coconut milk, 20g of green chili, 20g of red onion and 10g of salt and served for a total of 30 panelists. They ranked the samples for five parameters: color, aroma, texture, bitterness and overall preference using a three-tier scoring system (the highest, medium and lowest levels of choice were indicated by a score of three, two and one, respectively for each parameter). The generated data were subjected to FREQ procedure in SAS to decipher the associations with varietal preferences. For each *Luffa* variety, a weighted score was calculated by multiplying raw percentages with the associated rank for each taste parameter. Thereby weighted scores were assigned separately for color, aroma, texture, bitterness and overall preference for each variety. The weighted scores were subjected to PCA using Minitab, and the PC biplot between two major PCs (PC1 and PC2), Eigenvalue plot and the Scree plot were obtained to depict the contribution of each organoleptic parameter to the total variance of preference among eight *Luffa* varieties.

## Results

### Variation of the vegetative parameters

**Fig 1:**
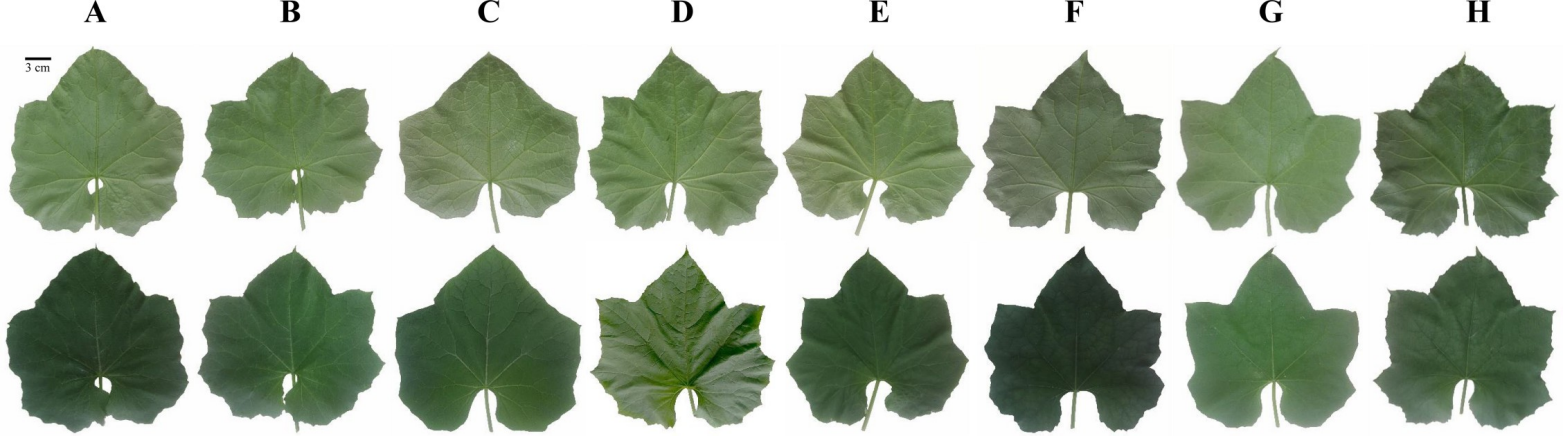
Morphological variation of abaxial (upper row) and adaxial (lower row) surfaces of the leaves of two *Luffa* spp. A: *Asiri*; B: *Gannoruwa Ari* (GA); C: LA33; D: *Naga-F1*; E: *Nadee- F1*, F: *Niyan Watakolu* Yellow Peel (NWYP); G: *Niyan Watakolu* Green Peel (NWGP) H: LF3522.

The vegetative parameters assessed in the present study displayed high variance (Fig 1, Table 2). The two hybrids, *Naga-F1* and *Nadee-F1*, had the significantly highest LL (16.21 cm and 16.09 cm respectively) while LF3522, an *L*. *aegyptiaca* variety possessed the significantly lowest LL (9.63 cm) (*P*<0.001). The *L*. *aegyptiaca* varieties had significantly lowest LW. There was no significant difference between the varieties in PL. The NLS was significantly highest in NWYP and NWGP (58.25 and 52.50, respectively) (*P*<0.001). Significantly lower RDW values were recorded for *Asiri*, GA, LA33, *Naga-F1* and LF3522 (*P*<0.001). There was no noted difference in the RTL among the eight varieties.

**Table 2:** Variation of the vegetative traits.

Species	Variety	Leaf			Stem		Root	
		LL (cm)	LW (cm)	PL (cm)	INL (cm)	NLS	RTL (cm)	RDW (g)
*L*. *acutangula*	*Asiri*	11.50^c^ ±0.34	17.25^b^ ±0.23	11.70^a^ ±0.45	16.37^b^ ±0.31	35.51^b^ ±2.50	20.40^a^±0.38	17.00^b^±0.43
	GA	13.50^b^ ±0.42	16.82^b^ ±0.56	13.20^a^ ±0.40	17.87^b^ ±0.46	29.70^b^ ±2.70	19.82^a^±0.33	15.11^b^±0.26
	LA33	13.78^b^ ±0.59	17.10^b^ ±0.57	12.15^a^ ±0.40	14.36^c^ ±0.35	36.51^b^ ±4.50	18.30^a^±0.99	13.67^b^±0.40
	*Naga-F1*	16.21^a^ ±0.46	18.13^b^ ±0.78	12.30^a^ ±0.43	17.33^b^ ±0.40	30.90^b^ ±5.80	20.81^a^±0.70	16.06^b^±0.45
	*Nadee-F1*	16.09^a^ ±0.51	21.00^a^ ±0.59	11.90^a^ ±0.55	20.36^a^ ±0.35	40.80^b^ ±1.20	18.10^a^±0.90	23.19^a^±0.81
*L*. *aegyptiaca*	NWYP	10.57^d^ ±0.35	13.71^c^ ±0.28	10.80^a^ ±0.39	12.50^c^ ±0.26	58.25^a^ ±3.80	23.21^a^±0.64	26.42^a^±1.06
	NWGP	10.21^d^ ±0.25	13.55^c^ ±0.29	12.40^a^ ±0.47	12.13^c^ ±0.32	52.50^a^ ±2.50	22.00^a^±0.72	26.91^a^±0.90
	LF3522	9.63^d^ ±0.15	12.25^c^ ±0.26	12.10^a^ ±0.37	13.20^c^ ±0.14	23.75^b^ ±2.10	20.20^a^± 0.56	18.82^b^±0.64

Means denoted by the same letters within the column are not significantly different at *P*<0.05

### Variation of the reproductive parameters

**Fig 2:**
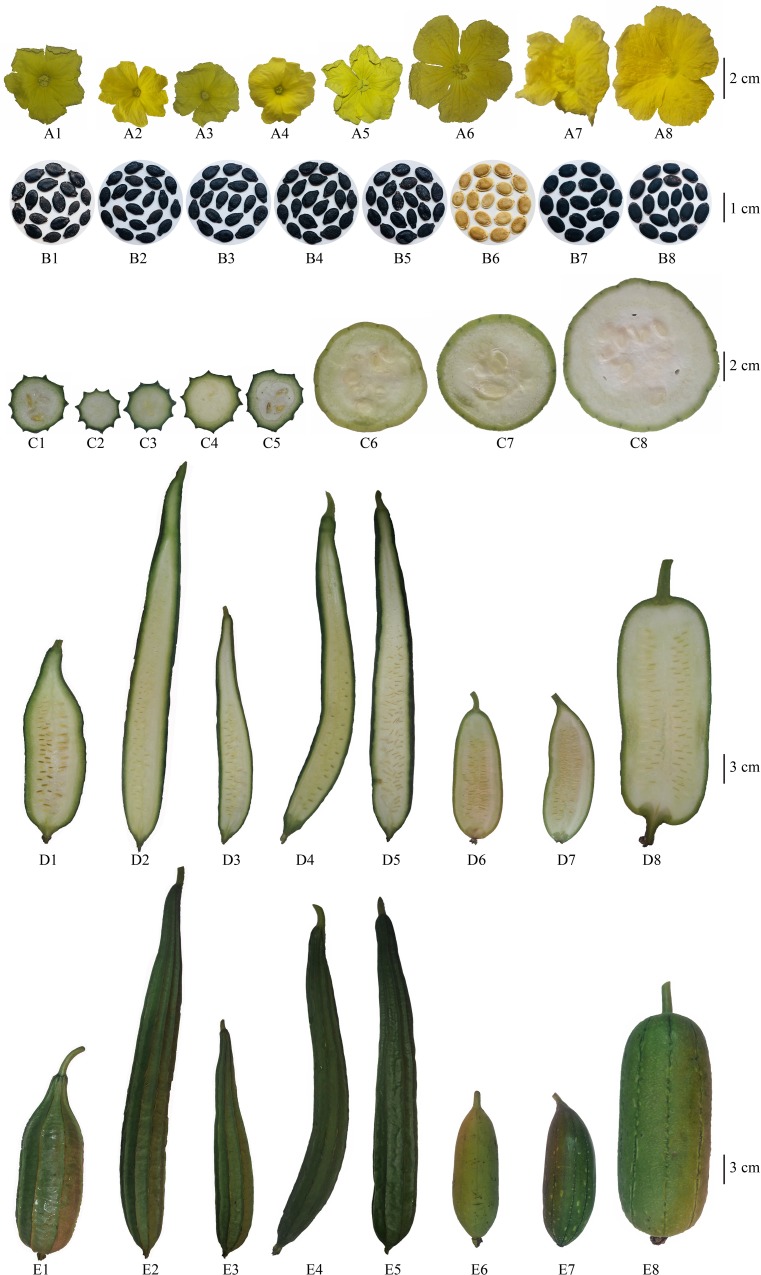
Morphological variation of the reproductive parts (A: Flowers; B: Seeds; C: Cross sections of fruits; D: Longitudinal sections of fruits; E: Whole fruit / external appearance). The numbers 1–8 indicate *Asiri*, *Gannoruwa Ari* (GA), LA33, *Naga-F1*, *Nadee-F1*, *Niyan Watakolu* Yellow Peel (NWYP), *Niyan Watakolu* Green Peel (NWGP), and LF3522 respectively.

The significant differences were observed for all the fruit parameters assessed (S1 Table) in the analysis except ST (*P*<0.001) (Table 3). NWGP and the NWYP had the significantly highest value for FN (29.77 and 23.94, respectively). Furthermore, the TW of NWGP and NWYP also recorded significantly highest values (6792.27 g and 6081.11 g, respectively). However, the varieties belonging to *L*. *acutangula*, the three local varieties (*Asiri*, GA and LA33) and the two exotic hybrids *Naga-F1* and *Nadee-F1* had significantly similar FT. The FT of the two *L*. *aegyptiaca* varieties were significantly different from each other. The FL was significantly lowest in NWGP, NWYP, and *Asiri* (Fig 2, S1 Fig). The ST of all the varieties of *Luffa* spp. indicated no significant differences (Fig 2, S1 Fig). The RN was also significantly similar among the fruits of five *L*. *acutangula* varieties while there were no ribs observed in the fruits of *L*. *aegyptiaca* varieties (Fig 2, S1 Fig). The HSW was significantly different among all the varieties (*P*<0.001). There were no significant differences observed among the eight varieties for SL, SW and NP (*P*<0.001) (Table 3, Fig 2).

**Table 3:** Variation of the reproductive traits.

Species	Variety	Fruits							Seeds					
		FN	FL (cm)	FW (cm)	FG (cm)	RN	ST (cm)	FT (cm)	TW (g)	SL (mm)	SW (mm)	SET (mm)	HSW (g)	NP
*L*. *acutangula*	*Asiri*	3.53^b^ ±0.96	18.19^a^ ±0.72	3.89^c^ ±0.14	15.91^b^ ±0.42	10±0.00	0.39^a^ ±0.03	3.67^d^ ±0.16	465.01^c^ ±169.21	11.47^a^ ±0.29	6.56^a^ ±0.22	2.47^b^ ±0.07	15.95^e^ ±0.12	127.00^a^ ±8.54
	GA	4.89^b^ ±1.00	32.31^b^ ±1.10	3.89^c^ ±0.17	16.19^b^ ±0.48	10±0.00	0.42^a^ ±0.02	3.49^d^ ±0.12	937.18^c^ ±234.27	12.08^a^ ±0.08	7.07^a^ ±0.07	2.53^b^ ±0.07	19.71^b^ ±0.18	179.50^a^ ± 7.82
	LA33	3.78^b^ ±0.85	34.17^b^ ±2.59	3.47^c^ ±0.16	17.34^b^ ±3.52	10±0.03	0.40^a^ ±0.03	3.21^d^ ±0.12	677.28^c^ ±182.37	11.85^a^ ±0.09	6.47^a^ ±0.05	2.73^b^ ±0.03	11.28^f^ ±0.36	127.00^a^ ±5.87
	*Naga-F1*	7.57^b^ ±0.84	39.41^c^ ±0.58	3.95^c^ ±0.08	16.50^b^ ±0.27	10±0.00	0.49^a^ ±0.03	3.39^d^ ±0.16	2044.07^b^ ±246.17	11.55^a^ ±0.10	6.61^a^ ±0.15	2.58^b^ ±0.04	19.77^b^ ±0.11	99.50^a^ ±3.91
	*Nadee-F1*	7.72^b^ ±0.91	40.19^c^ ±1.32	3.77^c^ ±0.08	16.34^b^ ±0.45	10±0.00	0.48^a^ ±0.01	3.39^d^ ±0.12	2083.72^b^ ±233.95	12.25^a^ ±0.09	7.47^a^ ±0.12	3.16^a^ ±0.05	23.51^a^ ±0.16	123.00^a^ ±8.19
*L*. *aegyptiaca*	NWYP	29.77^a^ ±1.84	18.52^a^ ±0.37	4.53^b^ ±0.09	18.20^b^ ±0.15	0	0.33^a^ ±0.02	4.28^c^ ±0.15	6792.27^a^ ±471.35	11.24^a^ ±0.16	7.84^a^ ±0.08	2.39^b^ ±0.05	17.61^c^ ± 0.10	199.00^a^ ±13.93
	NWGP	23.94^a^ ±2.92	17.95^a^ ±0.20	5.02^b^ ±0.05	18.69^b^ ±0.25	0	0.35^a^ ±0.01	4.82^b^ ±0.07	6081.11^a^ ±800.36	10.80^a^ ±0.10	7.64^a^ ±0.12	2.27^b^ ±0.04	16.85^d^ ± 0.11	200.00^a^ ±7.07
	LF3522	3.10^b^ ±0.40	29.75^b^ ±0.93	8.22^a^ ±0.29	30.49^a^ ±0.65	0	0.44^a^ ±0.03	8.08^a^ ±0.12	2875.39^b^ ±485.02	11.67^a^ ±0.27	7.24^a^ ±0.15	2.53^b^ ±0.06	16.03^e^ ±0.17	281.50^a^ ±10.77

Means denoted by the same letters within the column are not significantly different at *P*<0.05

The varieties NWYP, NWGP, and LF3522 of *L*. *aegyptiaca* and *Asiri* and LA33 of *L*. *acutangula* had taken the significantly highest number of days for the opening of male flowers (DMF) and highest number of days for the opening of female flowers (NFF) (Table 4, *P*<0.001). The two hybrids got the least number of days for the DHV (10–11 days) while all the three varieties of *L*. *aegyptiaca* got the significantly highest number of days for DHV (18–19 days) (Table 4). Furthermore, there was no apparent variation between the two species on the NDH. There was no significant difference among the varieties in the parameters; FFN, VLF, and VGF (Table 4).

**Table 4:** Variation of days in reaching flowering and harvesting stages.

Species	Variety	DMF	NFF	DHV	NDH	MFR	FFN	VLF (m)	VGF (cm)	PDL (cm)
*L*. *acutangula*	*Asiri*	43.00^a^ ±1.50	42.00^a^ ±1.60	16.00^b^ ±0.61	43.23^a^ ±0.98	11.25^a^ ±0.3	10.49^a^±0.34	1.67^a^±0.06	2.13^a^±0.04	4.40^f^±0.11
	GA	37.00^b^ ±1.05	33.50^b^ ±0.52	14.00^b^ ±0.47	36.82^b^ ±0.47	13.19^a^ ±1.3	6.48^a^±0.23	2.34^a^±0.09	2.00^a^±0.07	5.60^d^±0.15
	LA33	45.00^a^ ±1.81	42.50^a^ ±1.20	15.50^b^ ±1.12	45.28^a^ ±0.67	13.42^a^ ±1.5	5.48^a^±0.28	2.82^a^±0.12	2.65^a^±0.08	4.10^f^±0.33
	*Naga-F1*	31.00^b^ ±0.44	31.50^c^ ±0.42	10.50^c^ ±0.35	31.00^b^ ±0.37	15.49^a^ ±0.0	5.48^a^±0.26	2.64^a^±0.11	2.31^a^±0.04	7.00^c^±0.23
	*Nadee-F1*	33.50^b^ ±0.48	30.50^c^ ±0.43	11.00^c^ ±0.54	33.09^b^ ±0.33	5.74^b^ ±0.3	6.93^a^±0.19	3.01^a^±0.11	2.08^a^±0.04	7.10^c^±0.10
*L*. *aegyptiaca*	NWYP	44.00^a^ ±1.25	43.50^a^ ±0.85	19.00^a^ ±0.65	43.84^a^ ±0.74	5.00^b^ ±0.5	7.75^a^±0.33	2.53^a^±0.10	2.41^a^±0.06	8.26^b^±0.36
	NWGP	48.50^a^ ±1.27	49.00^a^ ±0.67	18.00^a^ ±0.31	48.00^a^ ±0.84	4.09^b^ ±0.4	14.87^a^±0.38	3.18^a^±0.20	2.42^a^±0.09	4.94^e^±0.18
	LF3522	43.00^a^ ±0.95	46.50^a^ ±1.23	19.50^a^ ±0.71	42.89^a^ ±0.59	3.46^b^ ±0.5	12.41^a^±0.42	1.94^a^±0.12	2.09^a^±0.08	13.97^a^±0.53

Means denoted by the same letters within the column are not significantly different at *P*<0.05

### Morphological diversity structure

**Fig 3:**
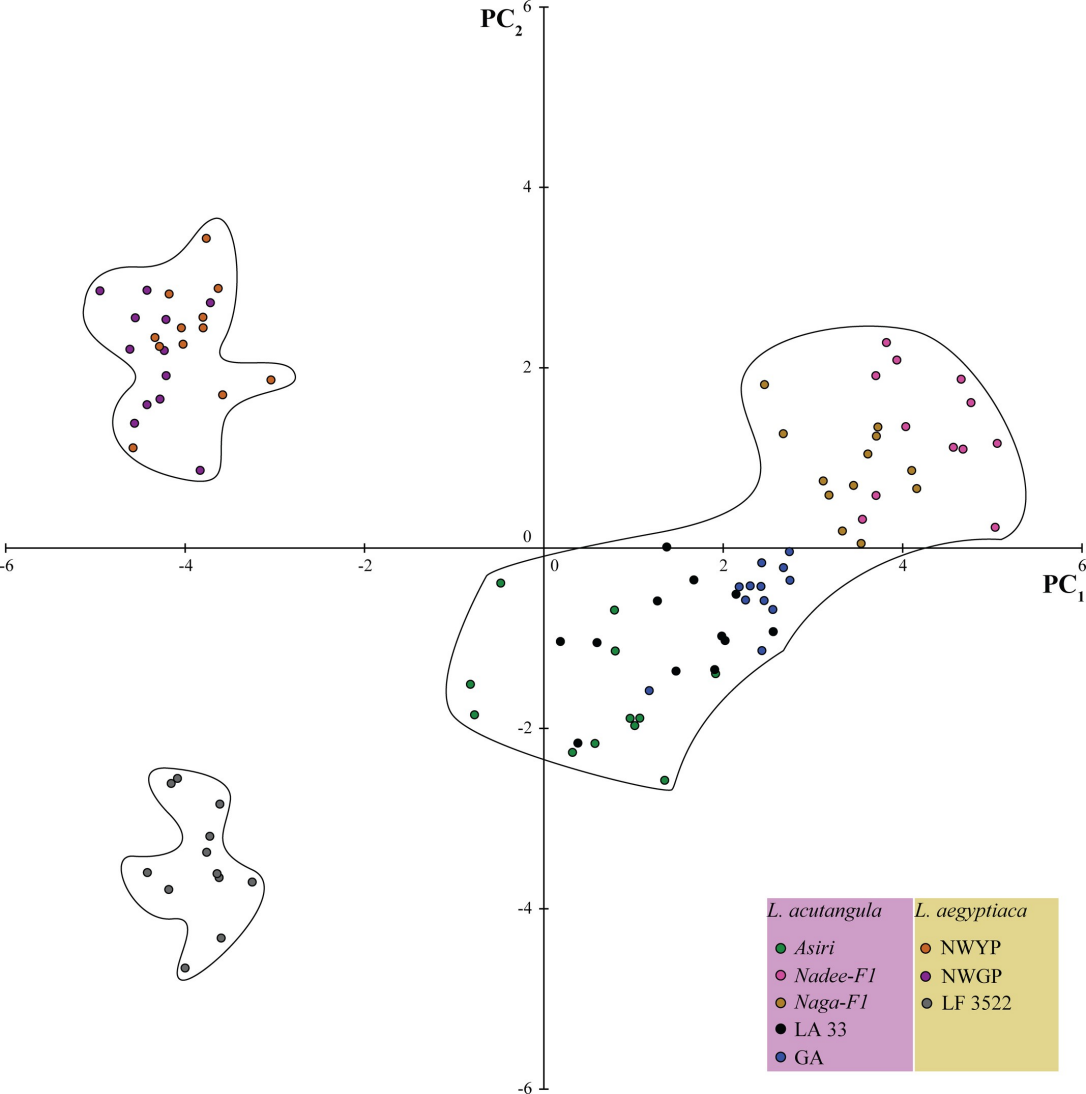
PC biplot for the varieties of *Luffa* spp. derived from the combined PCA of reproductive and vegetative parameters. Three distinct clusters were obtained.

**Fig 4:**
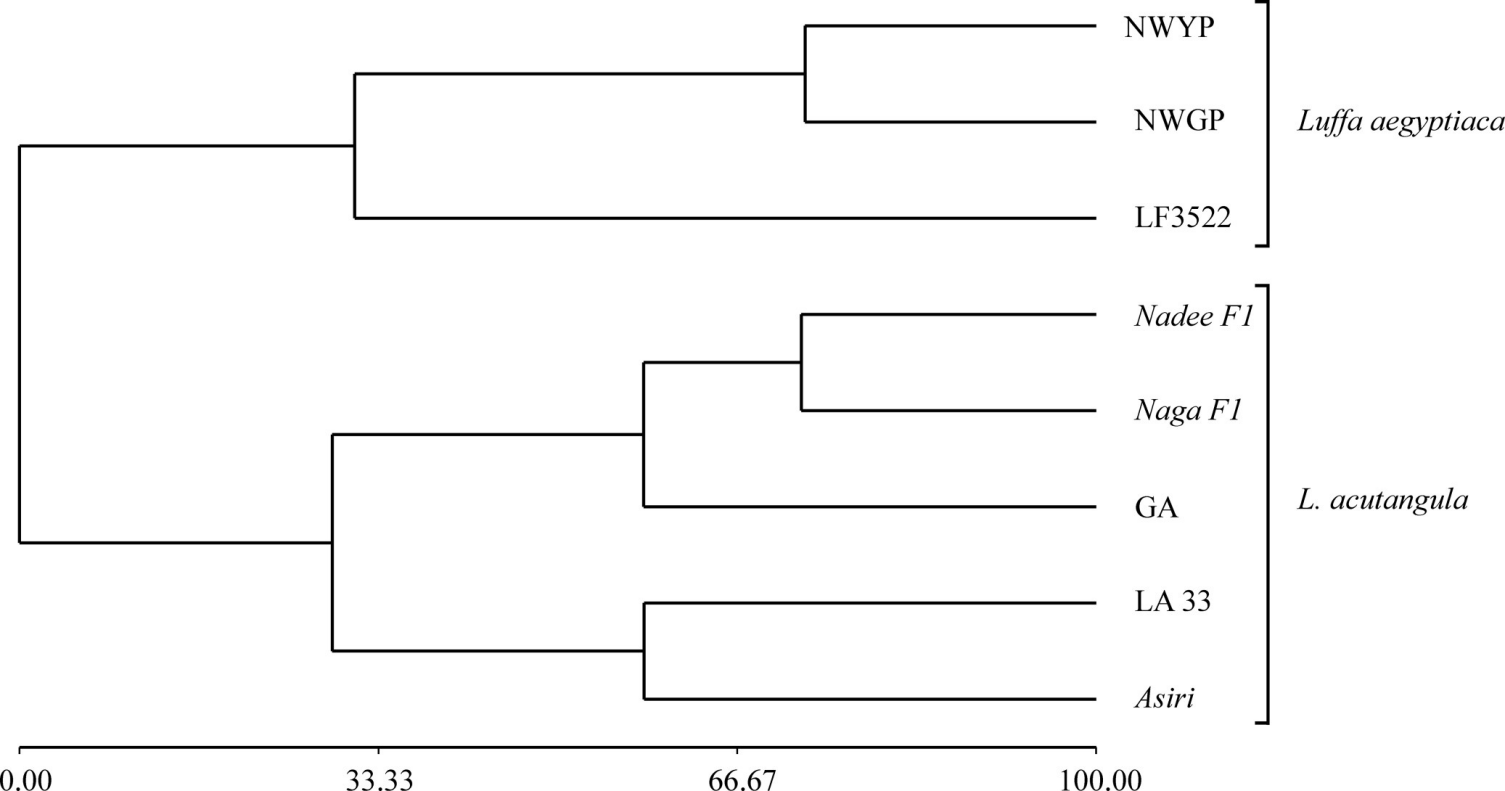
Dendrogram constructed for eight varieties of the two *Luffa* spp. based on principal components calculated from vegetative and reproductive characters, using Complete Linkage and Euclidean Distance.

The PCA yielded 32 principal components (PCs) (S4 Table) collectively for the vegetative and reproductive parameters. In the PC biplot between PC1 and PC2, the eight varieties were grouped into three discrete clusters (Fig 3). All the varieties belonging to *L*. *acutangula* were clustered together, at 29.04% similarity coefficient as revealed in the dendrogram generated using the PCs (Fig 4). However, the three *L*. *aegyptiaca* varieties were split into two main clusters where the two land races of *L*. *aegyptiaca* were grouped together, and the LF3522 formed a separate cluster (Fig 4).

The dendrogram developed using Complete Linkage, and Euclidean Distance based on the principal components (S4 Table; S2A Fig) showed that all the five varieties of *L*. *acutangula* were fallen into a single cluster at 53% morphological similarity coefficient. However, the *L*. *aegyptiaca* variety LF3522 can be observed as an out-group although NWYP and NWGP clustered together at 68% morphological similarity coefficient. The two-hybrid varieties of *L*. *acutangula* (*Nadee-F1* and *Naga-F1*) showed a close similarity at 71.04% of the morphological similarity coefficient (Fig 4).

### Phylogenetic analysis

**Fig 5:**
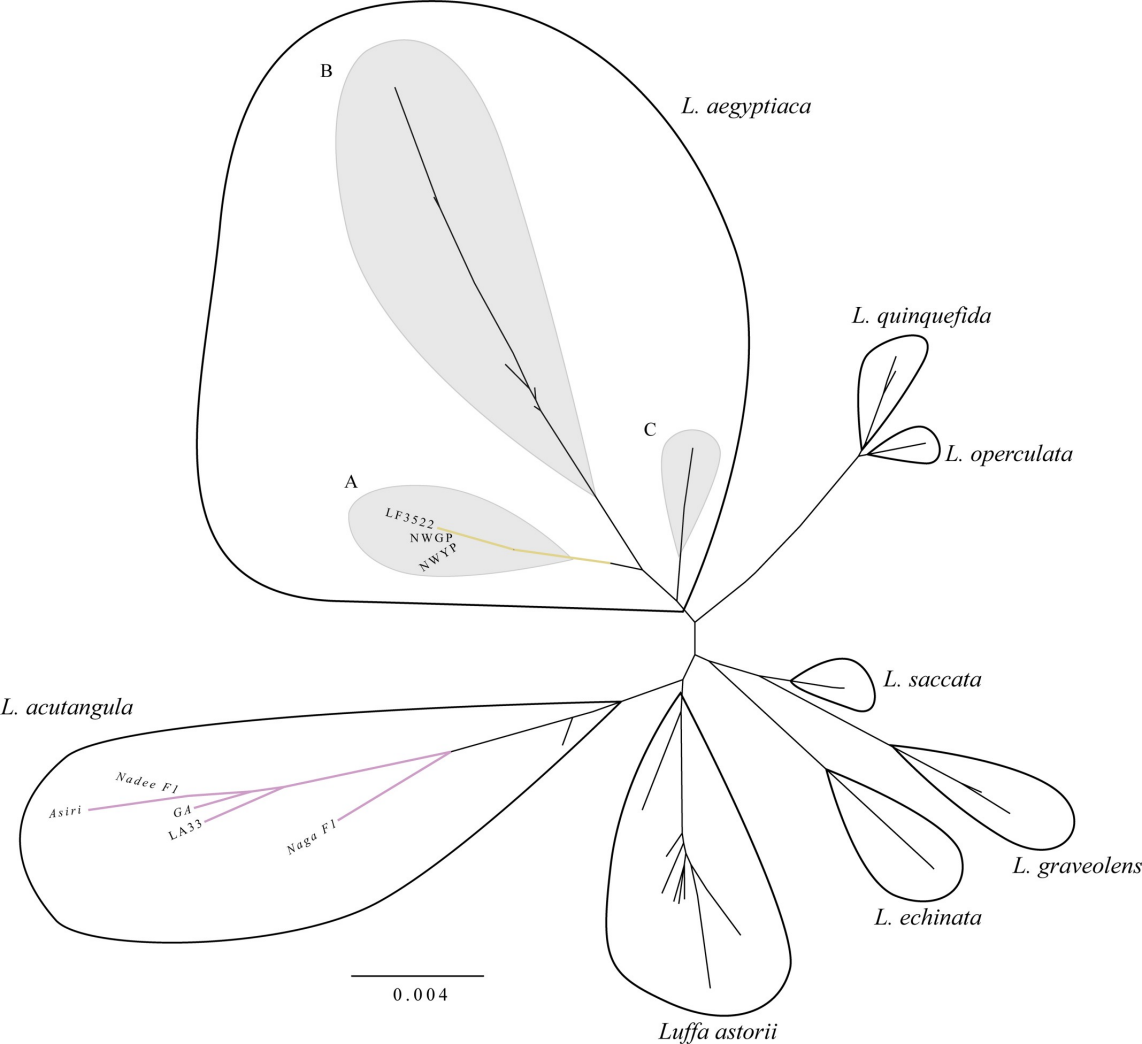
The unrooted Neighbour-Joining (NJ) tree constructed using combined datasets of *ITS*, *rbcL* and *trnH*-*psbA* markers. Each cluster contains different accessions of similar species as indicated next to the cluster. The *L*. *acutangula* and *L*. *aegyptiaca* clades that represent Sri Lankan varieties are highlighted in purple and yellow respectively. The three divergent clusters obtained within *L*. *aegyptiaca* clade are shaded.

The PCR products obtained for the markers; *trnH-psbA*, *rbcL*, *trnS*^*GCU*^*-trnG*^*UUC*^, *atpB-rbcL*, *matK-trnT*, *ITS*, *atpB gene*, *trnL-trnF*, and *trnL*, are depicted in S3 Fig. Out of these markers, we sequenced *ITS*, *rbcL* and *trnH*-*psbA*. The NJ and Bayesian trees had an almost similar topology. Because the NJ tree was more resolved, we presented it as an unrooted tree diagram (Fig 5). In the NJ tree, eight *Luffa* spp. separated into eight clades. The *L*. *aegyptiaca* and *L*. *acutangula* species sequenced during the present study were clustered as expected in the respective clades. Three well supported clades were visible in *L*. *aegyptiaca* cluster. The Clade A was exclusively consisting Sri Lankan *L*. *aegyptiaca* varieties that we sequenced [Boostrap values (bs) = 92, Posterior Probability (PP) = 99]. The Clade B contained the *L*. *aegyptiaca* accessions from Australian, South East Asian and South Asian countries (bs = 100, PP = 100). The Clade C included the *L*. *aegyptiaca* accessions from South East Asia and Australian regions (PP = 80). Thus Sri Lankan *L*. *aegyptiaca* sequences were diverged out from the Clades B and C of *L*. *aegyptiaca*. Moreover, *L*. *acutangula* varieties we sequenced, cladded in the cluster that contained *L*. *acutangula* from the other regions of the world (PP = 92). The sequence profile revealed a clear separation between *L*. *acutangula* and *L*. *aegyptiaca* with 1.4% of the genetic distance. The UPGMA also revealed the nesting of two landraces; NWYP and NWGP within the clade of *L*. *aegyptiaca* with a genetic distance of 0.14%, thus clearly separating from LF3522.

**Fig 6:**
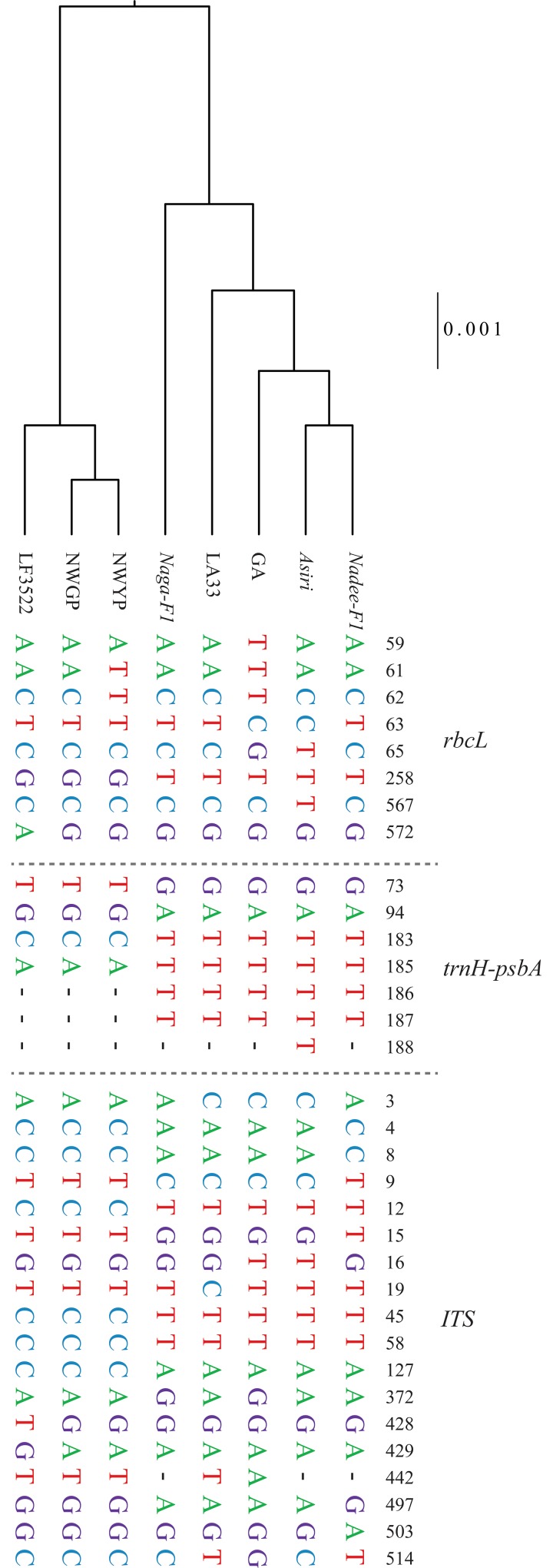
The SNP and INDEL profile of the eight varieties belonging to *L*. *acutangula* and *L*. *aegyptiaca*. Name of the marker and the position of the mutation acquired are given next to the alignment. A high genetic diversity could be observed among the varieties and thus enabled the identification of eight distinct haplotypes based on 30 SNPs and four INDELs present within the nucleotide and plastid genomic regions (A). The UPGMA dendrogram drawn using uncorrected pairwise distances of combined datasets of *ITS*, *rbcL* and *trnH*-*psbA* markers. The scale bar represents the percentage of genetic distance (B).

The nucleotide variations in nuclear and plastid regions derived from the sequence alignment of *Luffa* varieties are depicted in Fig 6. Accordingly, eight distinct haplotypes were identified. *ITS* was the most informative among the regions we sequenced due to the highest number of variable regions present. The sequence data was sufficient to capture the variance between the two landraces; NWYP and NWGP.

### Organoleptic assessment on *Luffa* fruits as a vegetable

The organoleptic properties evaluated through association analysis indicated a significant association between each variety of *Luffa* spp. and the taste parameters assessed except bitterness. A significant association was observed between the *Luffa* variety and color (S4A Fig, χ^2^ = 118.06), *Luffa* variety and the aroma (S4B Fig, χ^2^ = 44.32), *Luffa* variety and the texture (S4C Fig, χ^2^ = 59.05), *Luffa* variety and the overall preference (S4E Fig, χ^2^ = 95.23). There was no significant association between the variety and the bitterness at *P*<0.05 (S4D Fig).

The highest level of preference regarding color was recorded for *Naga-F1* while the least was recorded for NWYP (S4A Fig). *Naga-F1* further recorded a significantly higher preference for aroma. However, LA33 indicated the highest preference for aroma. *Nadee-F1*, NWGP, and LF3522 received the lowest preference for their aroma (S4B Fig). Furthermore, *Naga-F1* had the highest preference level for the texture compared to the other varieties. NWGP recorded the least preference regarding the texture (S4C Fig). *Asiri* had the highest level of overall preference whereas the NWGP had the lowest overall preference (S4E Fig).

**Fig 7:**
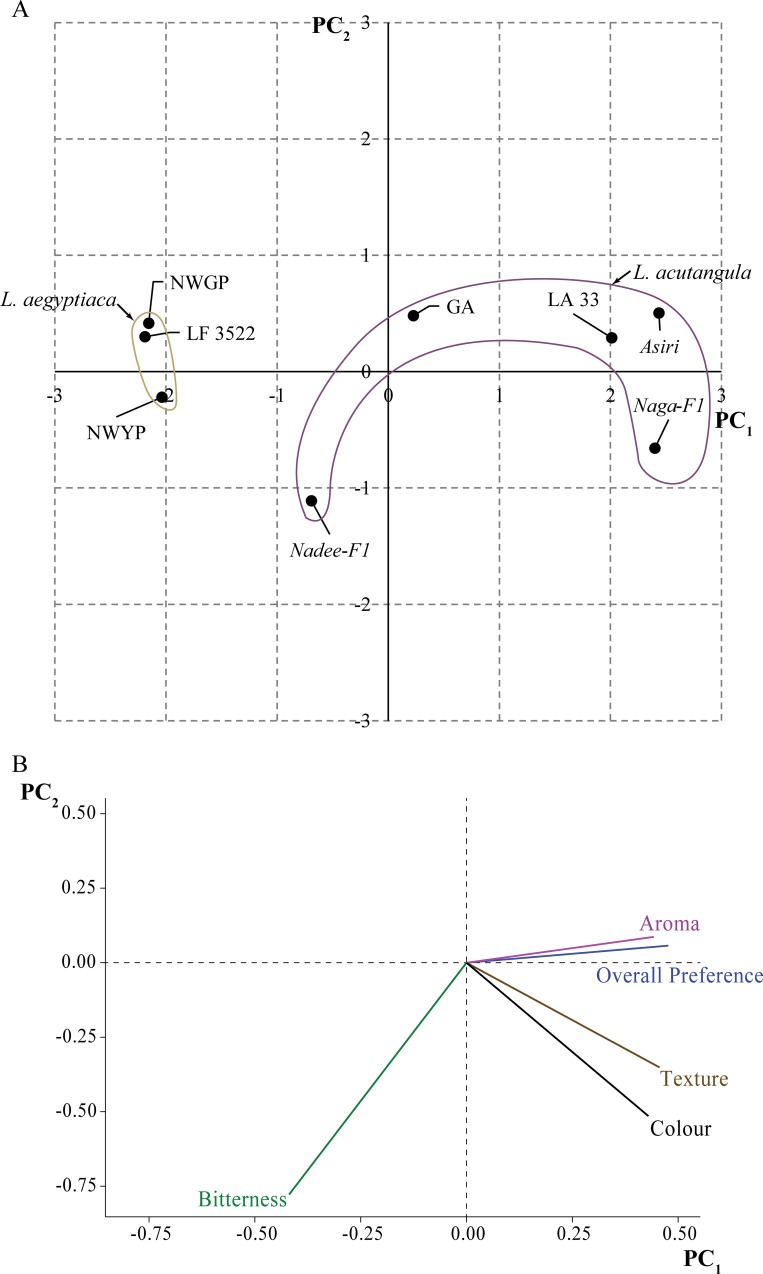
The PC biplot (A) and the Scree plot (B) drawn for weighted scores of the taste panel data.

The PC biplot constructed based on the PCA for organoleptic parameters indicated the highest preference values for *Naga-F1*, *Asiri*, LA33, and GA. The least preferences were indicated for *Nadee-F1* and the three *L*. *aegyptiaca* varieties; NWYP, NWGP, and LF3522. The PC biplot also divided the two species into clusters where all the *L*. *acutangula* varieties were clustered separately from the *L*. *aegyptiaca* varieties that were clustered together (Fig 7A). The loading plot constructed for the organoleptic parameters depicts that the overall preference for *Luffa* varieties was most closely associated with the aroma. The overall preference was not dependent on the bitterness of each of the variety (Fig 7B; S4D Fig).

## Discussion

### Variation of the vegetative parameters

The statistical analysis of the leaf traits indicated that LL and LW are the decisive traits that can be used to distinguish the two species (Fig 1). The mean LL and LW of the three *L*. *aegyptiaca* varieties; NWYP, NWGP, and LF3522 were markedly different from the varieties of the *L*. *acutangula* and showed comparatively lower values for the two leaf traits (*P*<0.001). Thus, we could identify that the significantly lower LL and LW of *L*. *aegyptiaca* varieties can be employed as morphological parameters in distinguishing the *Niyan Watakolu* varieties from the cultivated varieties of *L*. *acutangula*. Further, analysis on the leaf morphology and leaf texture indicated that the *L*. *acutangula* has smoothly textured adaxial and abaxial surfaces which are shallowly lobed. The *L*. *aegyptiaca* had contrasting features where the leaves were rough in texture with deep and prominent lobes (Fig 1). These features are consistent with the prior records on the Indian *Luffa* varieties [4]. The NLS of the two *Niyan Watakolu* varieties were significantly different from all the other varieties further suggesting their uniqueness from the other varieties of *Luffa* spp. However, INL and the two root traits (RTL and RDW) cannot be employed as vegetative parameters for species delimitation.

*L*. *aegyptiaca* varieties, NWYP and NWGP were significantly similar to each other in all the vegetative parameters reported in the present study. However, the two varieties did not indicate a clear difference from the other varieties in terms of the vegetative parameters. The varieties of *L*. *aegyptiaca* shared significant similarities with the varieties of *L*. *acutangula* for the majority of the vegetative traits such as PL, INL, RTL, and RDW. Thereby, statistical analysis of the vegetative parameters revealed that only the vegetative parameters LL and LW could be employed in the identification of the NWGP and NWYP from the other varieties of *Luffa* spp. (Table 2) inferring that the vegetative parameters do not provide sufficient evidence to reveal the species delimits and varietal identification of *Luffa* spp.

### Variation of the reproductive parameters

We identified that NWGP and NWYP recorded a significant difference in the fruit traits FN, FW and TW in comparison to all other *Luffa* varieties. The reproductive parameters can be successfully utilized in distinguishing of two *L*. *aegyptiaca* varieties; NWGP and NWYP from the other *Luffa* varieties. Significantly similar values obtained for ST suggest that it cannot be used as a fruit trait in the identification of NWGP and NWYP. The relatively similar ST values for each variety is indicated in (S1A–S1E Fig) by the cross sections of each variety of *Luffa* spp. However, none of the seed traits such as SL, SW, SET, NP, and HSW can be employed for the identification of NWGP and NWYP from the other varieties. The reproductive parameters at the flowering and harvesting stages also indicate certain traits that are of relative significance regarding the species identification (Table 4). The parameters; DMF, NFF, DHV, and NDH, are relatively higher for the *L*. *aegyptiaca* varieties than the *L*. *acutangula* varieties which are relatively disadvantageous in terms of improving the crop potential of NWGP and NWYP.

Nevertheless, the other reproductive stage parameters; MFR, NFF, VLF, and VGF cannot be exploited in setting up species delimits as there is no significant difference between the two species. The significant difference in PDL for the two varieties, NWGP and NWYP, allows the successful differentiation between them. The overall analysis of the reproductive parameters indicated that out of the fruit, seed, flowering and harvesting stage traits, only FN, FW, and TW can be used in the identification of NWGP and NWYP from the other varieties, highlighting the limited applicability of morphological parameters.

### Combined analysis of vegetative and reproductive parameters

The vegetative and reproductive parameters did not provide the resolution for the species delimitation. Thus, we further extended our study focusing on both the vegetative and reproductive data collectively for better identification of inter and intra-species variation. In PCA with all the morphometric data followed by clustering, a clear separation was evident between the two species and the grouping of NWGP and NWYP separately from LF3522. Our results conform with the previous findings [4, 5].

### Phylogenetic analysis

The unrooted NJ tree revealed the nesting of *Luffa* sequences generated in the present study into well-separated clades of *L*. *acutangula* and *L*. *aegyptiaca* thus confirming the genetic identity of the two species. Although, *L*. *aegyptiaca* is previously named as *L*. *cylindrica* in Dassanayake and Fosberg (1988) [10], here we synonymize this species as *L*. *aegyptiaca* [3]. Similarly, in a study conducted by An et al. (2017) [9], the cladding of *L*. *aegyptiaca* and *L*. *acutangula* into two well defined clusters was evident. However, the three clades appeared within the *L*. *aegyptiaca* cluster revealed the genetic divergence of our *L*. *aegyptiaca* varieties with respect to a set of different accessions of *L*. *aegyptiaca* in different geographical regions in the world, as previously reported by Filipowicz et al. (2014) [3]. Thus, it is possible that Sri Lankan *L*. *aegyptiaca* could be a subspecies of *L*. *aegyptiaca*, which could be a unique genetic form naturalized in Sri Lanka. It is interesting to see the existence of a reciprocal monophyletic group within *L*. *aegyptiaca* clade (with the node support values of, bs = 92 and PP = 99). The *L*. *aegyptiaca* in Sri Lanka is known to be the landrace and an underutilized crop for which hybridization attempts have been implemented recently by Regional Agriculture Research and Development Center, Sri Lanka. It is also visible that the difference exists between landraces and the hybrid of *L*. *aegyptiaca* in vegetative and reproductive parameters. The similar pattern is also observed in our phylogenetic analysis (Fig 5 and Fig 6) indicating that the landrace could be a unique genetic form. The LF3522 had a slight divergence from *L*. *aegyptiaca* landraces mainly because of the hybridization of exotic varieties with NWGP and NWYP. LF3522 was clustered separately from NWGP and NWYP in the combined morphological and phylogenetic diversity structures. In Fig 3, LF3522 got separately clustered from NWGP/NWYP indicating the exotic origin. However, the exact origin of LF3522 is not available. Due to the reciprocal monophyly observed within the NJ tree (Fig 5), it is possible that LF3522 could have been originated due to a hybridization event between an exotic parent and a Sri Lankan variety of *L*. *aegyptiaca*.

We selected three barcoding markers, *ITS*, *rbcL*, and *trnH*-*psbA* to assess the two species of *Luffa*, due to the ease of amplification, clear banding pattern (S3 Fig) and the polymorphism reported in literature [3,54]. According to the SNP/INDEL profile, we identified *trnH-psbA* as the most reliable and desirable DNA barcode due to its high inter-specific sequence variation and non-ambiguous nature, further verifying high efficacy of *trnH-psbA* in the identification of species delimits which was also reported in Kress and Erickson (2007) [55].

### Organoleptic assessment

The association analysis of the organoleptic properties reveals that the NWGP and NWYP are the least preferred choices with respect to color, aroma, texture and overall preference (S4A, S4B, S4D and S4E Fig; respectively). Higher preference for the variety *Asiri* and the two hybrids *Naga*-*F1* and *Nadee*-*F1* suggests that the breeding protocols have played a key role in enhancing the organoleptic properties. We suggest that these varieties and the exotic hybrids have higher consumer preference due to the varietal improvements through breeding. The studies show that the consumers in India prefer both types of *Luffa* spp. in their immature forms regardless of the fruit size [56]. The organoleptic properties are more varied in terms of the culinary patterns of the different regions of the world. The Sri Lankan perspective with reference to the *L*. *aegyptiaca* varieties is contrasting compared to the organoleptic preferences of the other regions of the world. For instance, in Vietnam, *L*. *aegyptiaca* is highly preferred by the consumers for its inherent aroma, and thus cultivars with enhanced aroma and taste are being bred [57]. Similar approaches can be utilized in the improvement of the NWGP and NWYP varieties by cross-pollination with the varieties with the range of aroma and taste preferred by the Sri Lankan consumers. The PC biplot created for the variation of the organoleptic parameters based on PCA analysis led to the close clustering of *L*. *aegyptiaca* varieties together which received lower preference scores compared to the varieties of *L*. *acutangula* (Fig 7A, S2B Fig, S5 Table). The *L*. *acutangula* varieties got dispersedly positioned indicating that *Asiri*, GA, LA33, *Naga-F1*, and *Nadee-F1* are preferred highly compared to NWYP, NWGP, and LF3522 (Fig 7A). The bitterness was not significantly associated with *Luffa* whereas all other four parameters were significantly associated. The Scree plot also depicts that the bitterness has a meager influence on the overall taste. The aroma is almost overlapped with overall taste with its position indicating that the aroma mostly determines the overall preference. The diversity structure created for organoleptic parameters demonstrates the need of improving *L*. *acutangula* varieties *Nadee-F1* and GA, and all the varieties of *L*. *aegyptiaca* for better consumer preference. Within *L*. *acutangula*, it is possible to make segregating populations between highly preferred and low preferred varieties as the parents to create segregating populations to detect the QTL for marker assisted breeding.

### Avenues for improving the crop potential

Despite the favorable traits that support the crop potential of NWGP and NWYP, we identified several reproductive traits that are undesirable. The comparatively higher DMF, DFF, DHV, and DHF values are relatively disadvantageous in their utilization as a crop species. Thus, the enhancement of the crop potential of NWGP and NWYP requires the shortening of the DFF, DMF, DHV, and DHF. Therefore, we suggest the implementation of breeding protocols for the NWGP and NWYP varieties for shorter harvesting periods. Furthermore, the outcomes of the organoleptic evaluation indicate a barrier for the utilization of the *L*. *aegyptiaca* as a crop. The higher preference values for the varieties and hybrids of *L*. *acutangula* suggest that the selection and breeding for varietal improvement has played a major role in enhancing the organoleptic properties. The current Sri Lankan varieties including GA are bred for higher yields [27]. However, the *L*. *aegyptiaca* varieties are not currently subjected to any varietal improvement procedures. Therefore, the organoleptic assessment results stress the importance of introgressing the preferred organoleptic traits to NWGP and NWYP. Similar studies have been conducted in other countries such as Vietnam to improve aroma. The breeding programs have been implemented in Vietnam to breed for *L*. *aegyptiaca* hybrids with subsequent selection for preferred levels of taste and aroma [55]. Similar approaches can be utilized in the improvement of the NWGP and NWYP varieties by cross pollinating with the varieties with the aroma and taste levels preferred by the Sri Lankan consumers.

## Conclusions

The combined data of vegetative and reproductive parameters classifies *L*. *acutangula* varieties into two distinct clusters at 29.04% of morphological similarity coefficient. The sequence polymorphism of *trnH-psbA* establishes the species delimits of *L*. *acutangula* and *L*. *aegyptiaca* where *L*. *acutangula* varieties have the GATTTT haplotype whereas *L*. *aegyptiaca* has the TGCA haplotype. The sequence polymorphism in *rbcL* establishes the varietal identification of *L*. *aegyptiaca* whereas *ITS* polymorphism establishes the varietal identities of *L*. *acutangula*. The phylogenetic analysis infers that the cultivated germplasm of *L*. *acutangula* forms a separate clade within the worldwide germplasm. The *L*. *aegyptiaca* varieties form a reciprocal monophyletic group with respect to other *L*. *aegyptiaca* germplasms found elsewhere in the world. *L*. *aegyptiaca* varieties studied in the present study could be identified as distinct genetic forms. The organoleptic assessment reveals that the aroma, texture, color, and overall preference are significantly different among the *Luffa* varieties assessed. Moreover, *L*. *aegyptiaca* varieties receive lower preference scores. The organoleptic parameters also differentiate two species where *L*. *aegyptiaca* varieties get tightly clustered. Therefore, the present study sets the species delimits, and the varietal identities based on the phylogenetic analysis and also shows the distinct morphological and organoleptic properties.

## Supporting information

S1 Fig The developing stages of the fruits of the varieties of *Luffa* spp. A: *Asiri*, B: *Gannoruwa Ari* (GA), C: LA33, D: *Naga*-*F1*, E: *Nadee-F1*, F: *Niyan Watakolu* Yellow Peel (NWYP), G: *Niyan Watakolu* Green Peel (NWGP), H: LF3522 varieties of *L*. *acutangula* and *L*. *aegyptiaca*. a→e indicate the developmental stages with three day gaps. The whole fruits are given in the top and the cross section are shown in the bottom. The scale bars indicate 3 cm.
(JPG)

S2 Fig Loading plot derived from PCs. A: Eigen values of the PCs derived from morphometric parameters, B: Eigen values of the PCs derived from weighted scores calculated for the organoleptic parameters.
(TIF)

S3 Fig The visualization of the PCR products of the DNA barcoding markers in 2.5% agarose gel electrophoresis. L: 50bp ladder. Positive controls [R1: Bg 366; R2: At 307 (two rice varieties) and A1: Spartan; A2: Tall cox (two apple varieties). Names of the nine DNA markers are indicated on the left. Approximate size of each band is marked on the right. The symbols *, #, $ and ∞ represent the specific bands in the increasing order of their sizes given in bps.
(TIF)

S4 Fig The evaluation of the associations between the variety and five sensory attributes; color, aroma, texture, bitterness and overall preference. Y-axis represent the percentage panelists responded in the survey. Pearson ᵪ2 and *P* value are indicated in the top right corner. The color of the bar with respect to the preferred level is given according to the key given bottom-right.
(JPG)

S1 Table List of abbreviations. (DOCX)

S2 Table Plant DNA barcoding markers, the PCR conditions, and the supporting references. (DOCX)

S3 Table List of *Luffa* species used in the study along with their geographic locations, voucher numbers and GenBank accession numbers. (DOCX)

S4 Table Eigen values and Covariances for the principal components of the combined morphometric parameters in PCA. (DOCX)

S5 Table Eigen values and Covariances for the principal components of the weighted scores of the organoleptic parameters in PCA. (DOCX)

## References

[1] Joshi BK, Tiwari RK, Ghale M., Gyawali S, Upadhyay MP. Nepalese landraces of sponge gourd for the production of tender fruits. JAE. 2013;14: 13–22. 10.3126/aej.v14i0.19782.

[2] Pandravada SR, Sivaraj N, Jairam R, Sunil N, Begum H, Reddy MT, et al *Luffa hermaphrodita*: First report of its distribution and cultivation in Adilabad, Andhra Pradesh, South India. Asian Agrihist. 2014;18(2): 123–132.

[3] Filipowicz N, Schaefer H, Renner SS. Revisiting *Luffa* (Cucurbitaceae) 25 years after C. heiser: species boundaries and application of names tested with plastid and nuclear DNA sequences. Syst Bot. 2014;39(1): 205–215. 10.1600/036364414X678215.

[4] Prakash K, Pandey A, Radhamani J, Bisht IS. Morphological variability in cultivated and wild species of *Luffa* (Cucurbitaceae) from India. Genet Resour Crop Evol. 2013;60(8): 2319–2329. 10.1007/s10722-013-9999-7.

[5] Cruz VMV, Tolentino MIS, Altoveros NC, Villavicencio MLH, Siopongco LB, dela Vitia AC, et al Correlations among accessions of Southeast Asian *Luffa* genetic resources and variability estimated by morphological and biochemical methods. Crop Sci. 1997;22(3): 131–140.

[6] Prakash K, Pati K, Arya L, Pandey A, Verma M. Population structure and diversity in cultivated and wild *Luffa* species. Biochem Syst Ecol. 2014; 56:165–170. 10.1016/j.bse.2014.05.012.

[7] Choudhary BR, Pandey S, Singh PK, Singh R. Genetic divergence in hermaphrodite ridge gourd (*Luffa acutangula*). Veg Sci. 2011;38(1): 68–72.

[8] Hoque S, Rabbani MG. Assessment of genetic relationship among landraces of Bangladeshi Ridge Gourd (*Luffa acutangula* Roxb.) using RAPD markers. J Sci Res. 2009;1(3): 615–623. 10.3329/jsr.v1i3.1968

[9] An J, Yin M, Zhang Q, Gong D, Jia X, Guan Y, et al Genome survey sequencing of *Luffa cylindrica* L. and microsatellite high resolution melting (SSR-HRM) analysis for genetic relationship of *Luffa* genotypes. Int J Mol Sci. 2017;18(9): 1942 10.3390/ijms18091942 28891982 PMC5618591

[10] Dassanayake MD, Fosberg FR. A revised handbook of the flora of Ceylon. Sri Lanka (SL): CRC Press; 1988.

[11] Heiser CB, Schilling EE. Phylogeny and distribution of *Luffa* (Cucurbitaceae). Biotropica. 1988;20(3): 185–191. 10.2307/2388233.

[12] Heiser CB, Schilling EE, Dutt B. The American species of *Luffa* (Cucurbitaceae). Syst Bot. 1988;13(1): 138–145. 10.2307/2419250

[13] Diaz MGQ, Ramirez DA. Cytogenetics of sponge gourd, *Luffa cylindrica* Roem., ridged gourd, *L*. *acutangula* Roxb., their F1 hybrid, F2 and BC 1 progenies. Philipp J Crop Sci. 1994; 2–6.

[14] Takahashi H, Suge H, Saito T. Sex expression as affected by N6-benzylaminopurine in staminate inflorescence of *Luffa cylindrica*. Plant Cell Physiol. 1980;21(4): 525–536. 10.1093/oxfordjournals.pcp.a076028.

[15] PROTA: Plant Resources of Tropical Africa. Wageningen, Netherlands. 2016. [cited 27 Oct 2018]. Available from: http://www.prota4u.org/.

[16] Beyer M, Hahn R, Peschel S, Harz M, Knonche M. Analysing fruit shape in sweet cherry (*Prunus avium* L.). Sci Hortic. 2002;96: 139–150. 10.1016/S0304-4238(02)00123-1.

[17] Zalapa JE, Staub JE, McCreight JD. Generation means analysis of plant architectural traits and fruit yield in melon. Plant Breed. 2006;125(5): 482–487. 10.1111/j.1439-0523.2006.01273.x.

[18] Kumar R, Ameta KD, Dubey RB, Pareek S. Genetic variability, correlation and path analysis in sponge gourd (*Luffa cylindrica* Roem.). Afr J Biotechnol. 2013;12(6): 539–543. 10.5897/AJB12.2968

[19] Okusanya OT, Ola-Adams BA, Bamidele JF. Variations in size, leaf morphology, and fruit characters among 25 populations of *Luffa aegyptiaca*. Can J Bot. 1981;59(12): 2618–2627. 10.1139/b81-314.

[20] Shah JJ, Thanki YJ, Kothari IL. Skeletal fibrous net in fruits of *Luffa cylindrica* M. Roem, and *Luffa acutangula* Roxb In: Nagaraj M, Malik CP, editors. Current trends in botanical research. New Delhi, India: Kalyani Publishers; 1980.

[21] Oboh IO, Aluyor EO. *Luffa cylindrica*- an emerging cash crop. Afr J Agric Res. 2009;4(8): 684–688.

[22] Manikandaselvi S, Vadivel V, Brindha P. Review on *Luffa acutangula* L.: Ethnobotany, phytochemistry, nutritional value and pharmacological properties. Int J Curr Pharm Res. 2016;7(3): 151–155.

[23] Akther F, Rahman A, Proma JJ, Kabir Z, Paul PK. Methanolic extract of *Luffa cylindrica* fruits show anti hyperglycemic potential in Swiss Albino mice. ANAS. 2014;8: 62–65.

[24] Yadav BS, Yadav R, Yadav RB, Garg M. Antioxidant activity of various extracts of selected gourd vegetables. J Food Sci. Technol. 2016;53(4): 1823–1833. 10.1007/s13197-015-1886-0 27413209 PMC4926887

[25] AgStat. Department of Agriculture; 2017. [cited 2018 Oct 29]. Available from: http://www.statistics.gov.lk/agriculture/ Agstat.

[26] Silva MWKP, Ranil RHG, Fonseka RM. *Luffa cylindrica* (L.) M. Roemer (Sponge Gourd- *Niyan wetakolu*): An emerging high potential underutilized cucurbit. Trop Agric Res. 2012;23(2): 186–191. 10.4038/tar.v24i2.8004.

[27] Bentota AP. Released and Recommended New Crop Varieties by the Varietal Release Committee of the Department of Agriculture Sri Lanka. Gannoruwa, Sri Lanka (SL): Department of Agriculture Press; 2013.

[28] Yadav RB, Chaudhary P, Khatiwada SP, Bajrachara J, Yadav RK, Upadhaya MP, et al Agro-morphological diversity of sponge gourd (*Luffa cylindrica* L.) in Bara, Nepal. On-farm management of agricultural biodiversity in Nepal. 2003: 42–47.

[29] Bajracharya JB, Rijal DK, Khatiwada SP, Paudel CL, Upadhaya MP, Pandey YR, et al. Agro-morphological characters and farmer perceptions: Data collections and analysis. In conserving agricultural biodiversity in situ: A scientific basis for sustainable agriculture Proceedings of workshop; Jul 5–12; Nepal; 1999.

[30] Schilling EE, Heiser CB. Flavonoids and the systematics of *Luffa*. Biochem Syst Ecol. 1981;9(4): 263–265. 10.1016/0305-1978(81)90006-5.

[31] Singh J, Dhall RK, Dhall P, Aulakh PS. Molecular characterization of biodiversity in vegetables -a review. Agric Rev. 2011;32(3): 193–201.

[32] Depatment of Agriculture. 2015. [cited 18 Oct 2018]. Available from: https://www.doa.gov.lk/HORDI/images/Document/English2015.pdf DOA.

[33] Rajapaksha RMSS, Siriwardana KPD, Ratnayake RHMK. Evaluation of Luffa (Luffa acutangula (L.) Roxb) varieties under low country intermediate zone of Sri Lanka. Proceedings of International Forestry and Environment Symposium, Sri Lanka; 2006.

[34] Dissanayake DRTNK, Herath HMSK, Gunadasa HKSG, Weerasinghe P. Nitrogen and potassium fertilizer response on growth and yield of hybrid Luffa–Naga-F1 variety. Proceedings of the Research Symposium of Uva Wellassa University; Jan 29–30. 2015.

[35] Panabokke CR. Soils and Agro-Ecological Environments of Sri Lanka. Natural Resources Series No. 2. Natural Resources Energy and Science Authority, 47/5, Maitland Place, Colombo 7, Sri Lanka. 1996.

[36] Joshi BK, Hari B KC, Tiwari RK, Ghale M, Sthapit BR, Upadhyay MP. 2004 Descriptors for sponge gourd (*Luffa cylindrica*). NARC, LIBIRD and IPGRI.

[37] Porebski S, Bailey LG, Baum BR. Modification of a CTAB DNA extraction protocol for plants containing high polysaccharide and polyphenol components. Plant Mol Biol Rep. 1997;15(1): 8–15. 10.1007/BF02772108.

[38] Wan CY, Wilkins TA. Spermidine facilitates PCR amplification of target DNA. Genome Res. 1993;3(3): 208–210. 10.1101/gr.3.3.208 8118404

[39] Sambrook J, Russell SW, editors. Molecular Cloning: A Laboratory Manual. 3. New York (NY): Cold Spring Harbor Laboratory Press; 2001.

[40] Kumar S, Stecher G, Tamura K. MEGA7: Molecular Evolutionary Genetics Analysis version 7.0 for bigger datasets. Mol Biol Evol. 2016;33(7): 1870–1874. 10.1093/molbev/msw054 27004904 PMC8210823

[41] Thompson JD, Higgins DG, Gibson TJ. CLUSTAL W: improving the sensitivity of progressive multiple sequence alignment through sequence weighting, position-specific gap penalties and weight matrix choice. Nucleic Acids Res. 1994;22(22): 4673–4680. 7984417 10.1093/nar/22.22.4673 PMC308517

[42] Vaidya G, Lohman DJ, Meier R. SequenceMatrix: concatenation software for the fast assembly of multi‐gene datasets with character set and codon information. Cladistics. 2011;27(2): 171–180. 10.1111/j.1096-0031.2010.00329.x. 34875773

[43] Planet PJ. Tree disagreement: measuring and testing incongruence in phylogenies. J Biomed Inform. 2006;39(1): 86–102. 10.1016/j.jbi.2005.08.008 16243006

[44] Swofford DL. PAUP, Phylogenetic Analysis using Parsimony (and other methods), Sinauer Associates, Sunderland; 2002.

[45] Stamatakis A. RAxML-VI-HPC: maximum likelihood-based phylogenetic analyses with thousands of taxa and mixed models. Bioinformatics. 2006;22(21): 2688–2690. 10.1093/bioinformatics/btl446 16928733

[46] Stamatakis A, Hoover P, Rougemont J. A rapid bootstrap algorithm for the RAxML web servers. Syst Biol. 2008;57(5): 758–771. 10.1080/10635150802429642 18853362

[47] Rodriguez FJ, Oliver JL, Marin A, Medina JR. The general stochastic model of nucleotide substitution. J Theor Biol. 1990;142(4): 485–501. 10.1016/S0022-5193(05)80104-3. 2338834

[48] Huelsenbeck JP, Ronquist F. MrBayes: Bayesian inference of phylogeny. Bioinformatics. 2001;17: 754–755. 10.1093/bioinformatics/17.8.754. 11524383

[49] Akaike H. A new look at the statistical model identification. IEEE Trans Automat Contr. 1974;19(6): 716–723. 10.1109/TAC.1974.1100705.

[50] Cavanaugh JE. Unifying the derivations for the Akaike and corrected Akaike information criteria. Stat Probabil Lett. 1997;33(2): 201–208. 10.1016/S0167-7152(96)00128-9.

[51] Posada D. jModelTest: phylogenetic model averaging. Mol Biol Evol. 2008;5: 1253–1256. 10.1093/molbev/msn083. 18397919

[52] Miller MA, Pfeiffer W, Schwartz T. 2010. Creating the CIPRES Science Gateway for inference of large phylogenetic trees. Gateway Computing Environments Workshop (GCE). [cited 21 Sep 2018]. Available from: http://www.ieeexplore.ieee.org/abstractdocument/5676129.

[53] Rambaut A. FigTree, a graphical viewer of phylogenetic trees. Version 1.4.3. 2014. [cited 2018 Sep 21]. Available from: http://tree.bio.ed.ac.uk/software/figtree.

[54] Pang X, Liu C, Shi L, Liu R, Liang D, Li H, et al 2012 Utility of the *trnH–psbA* intergenic spacer region and its combinations as plant DNA barcodes: a meta-analysis. PLoS ONE. 2012:7(11):e48833 10.1371/journal.pone.0048833. PMC3498263 23155412

[55] Kress WJ, Erickson DL. A two-locus global DNA barcode for land plants: the coding *rbcL* gene complements the non-coding *trnH-psbA* spacer region. PLoS one. 2007;2(6): 508 10.1371/journal.pone.0000508. PMC1876818 17551588

[56] Dhillon NPS, Sanguansil S, Singh SP, Masud MAT, Kumar P, Bharathi LK, et al Gourds: bitter, bottle, wax, snake, sponge and ridge In: Grumet R, Katzir N, Garcia-Mas J, editors. Genetics and Genomics of Cucurbitaceae. Plant Genetics and Genomics: Crops and Models, Springer, Cham; 2016 pp. 155–172.

[57] Truong HTH, Phan TT, Dieu TT, Nguyen. Evaluation of Sponge Gourd (*Luffa cylindrica* L.) Inbred Lines for Growth Potential and Fruit Quality in Thua Thien Hue Province, Central Vietnam. JAST. 2017;7: 10–16.

