# Supplementary figures and images for: The establishment of the species-delimits and varietal-identities of the cultivated germplasm of Luffa acutangula and Luffa aegyptiaca in Sri Lanka using morphometric, organoleptic and phylogenetic approaches
Source: PLoS One. 2019 Apr 9;14(4):e0215176. doi: 10.1371/journal.pone.0215176 (PMC6456250; doi:10.1371/journal.pone.0215176)

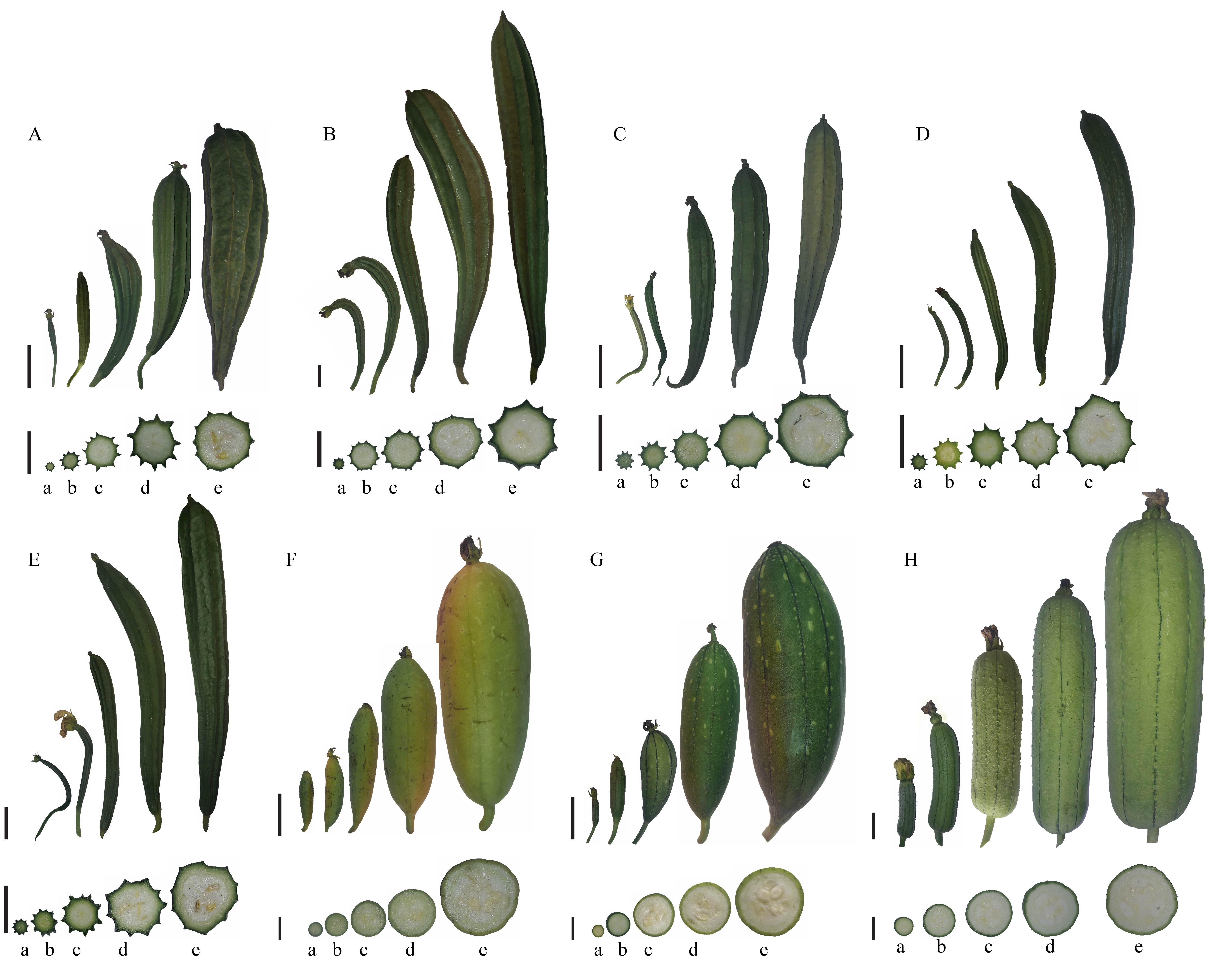

Supplement: S1 Fig — A: Asiri, B: Gannoruwa Ari (GA), C: LA33, D: Naga-F1, E: Nadee-F1, F: Niyan Watakolu Yellow Peel (NWYP), G: Niyan Watakolu Green Peel (NWGP), H: LF3522 varieties of L. acutangula and L. aegyptiaca. a→e indicate the developmental stages with three day gaps. The whole fruits are given in the top and the cross section are shown in the bottom. The scale bars indicate 3 cm. (JPG) [file pone.0215176.s001.jpg]

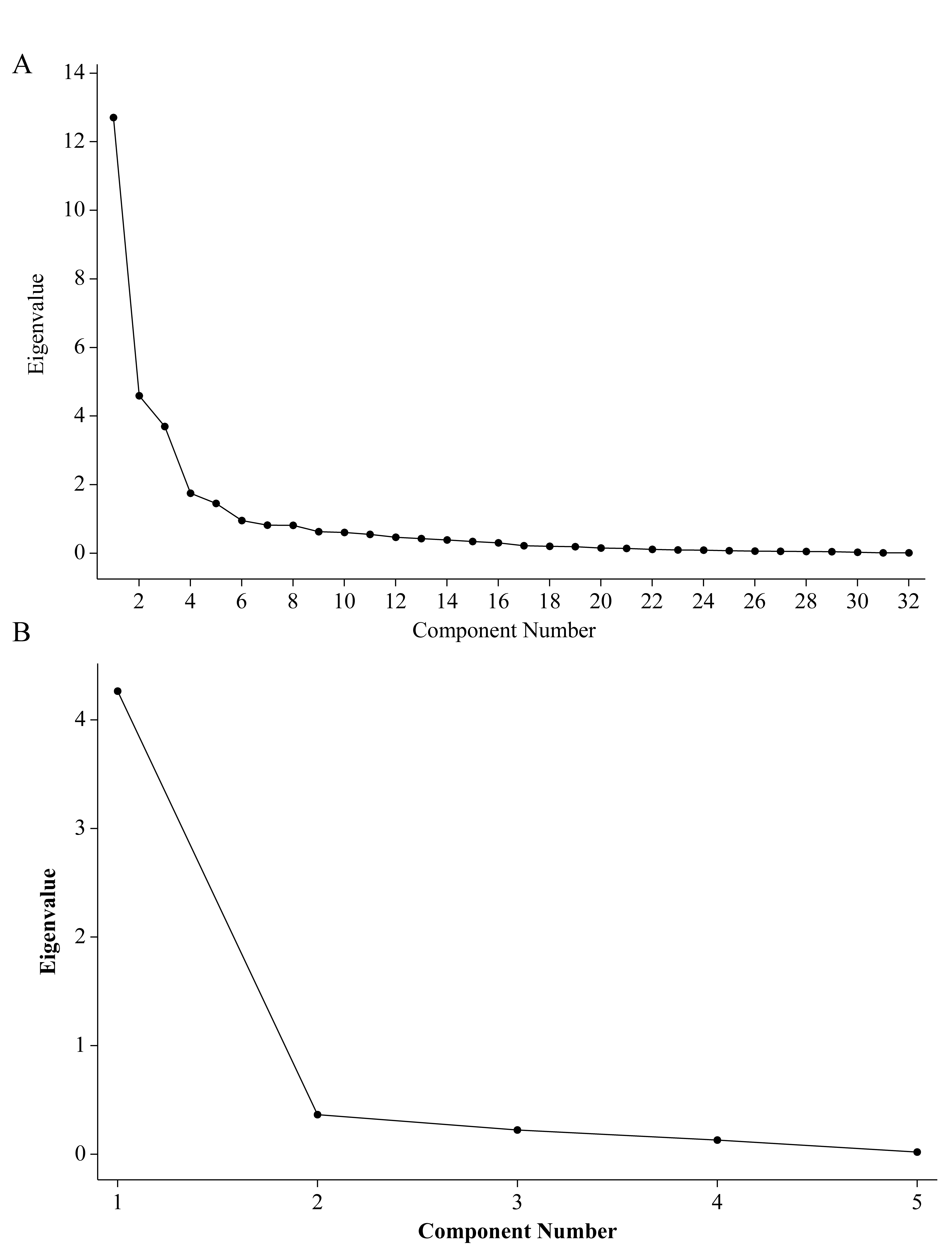

Supplement: S2 Fig — A: Eigen values of the PCs derived from morphometric parameters, B: Eigen values of the PCs derived from weighted scores calculated for the organoleptic parameters. (TIF) [file pone.0215176.s002.tif]

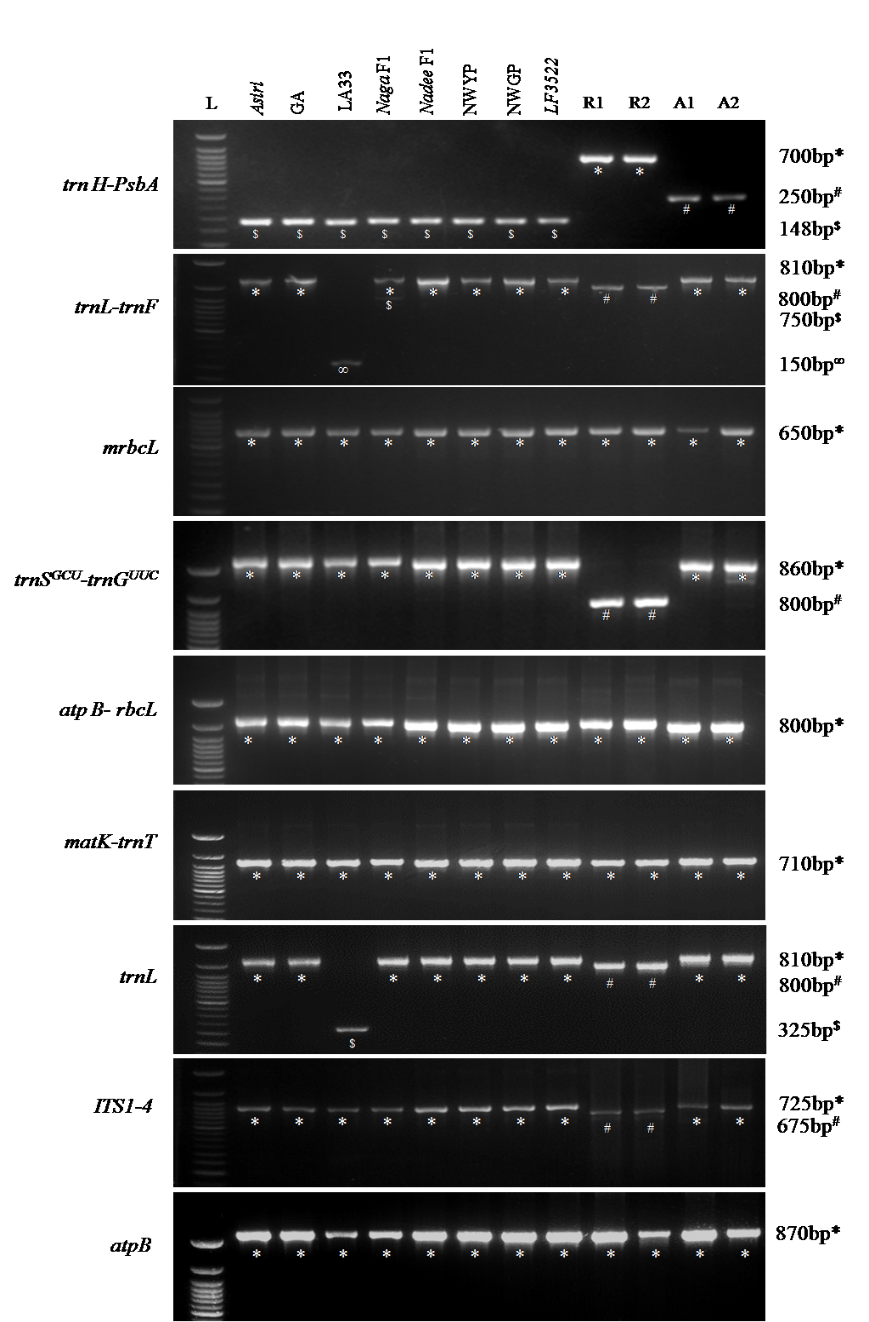

Supplement: S3 Fig — L: 50bp ladder. Positive controls [R1: Bg 366; R2: At 307 (two rice varieties) and A1: Spartan; A2: Tall cox (two apple varieties). Names of the nine DNA markers are indicated on the left. Approximate size of each band is marked on the right. The symbols *, #, $ and ∞ represent the specific bands in the increasing order of their sizes given in bps. (TIF) [file pone.0215176.s003.tif]

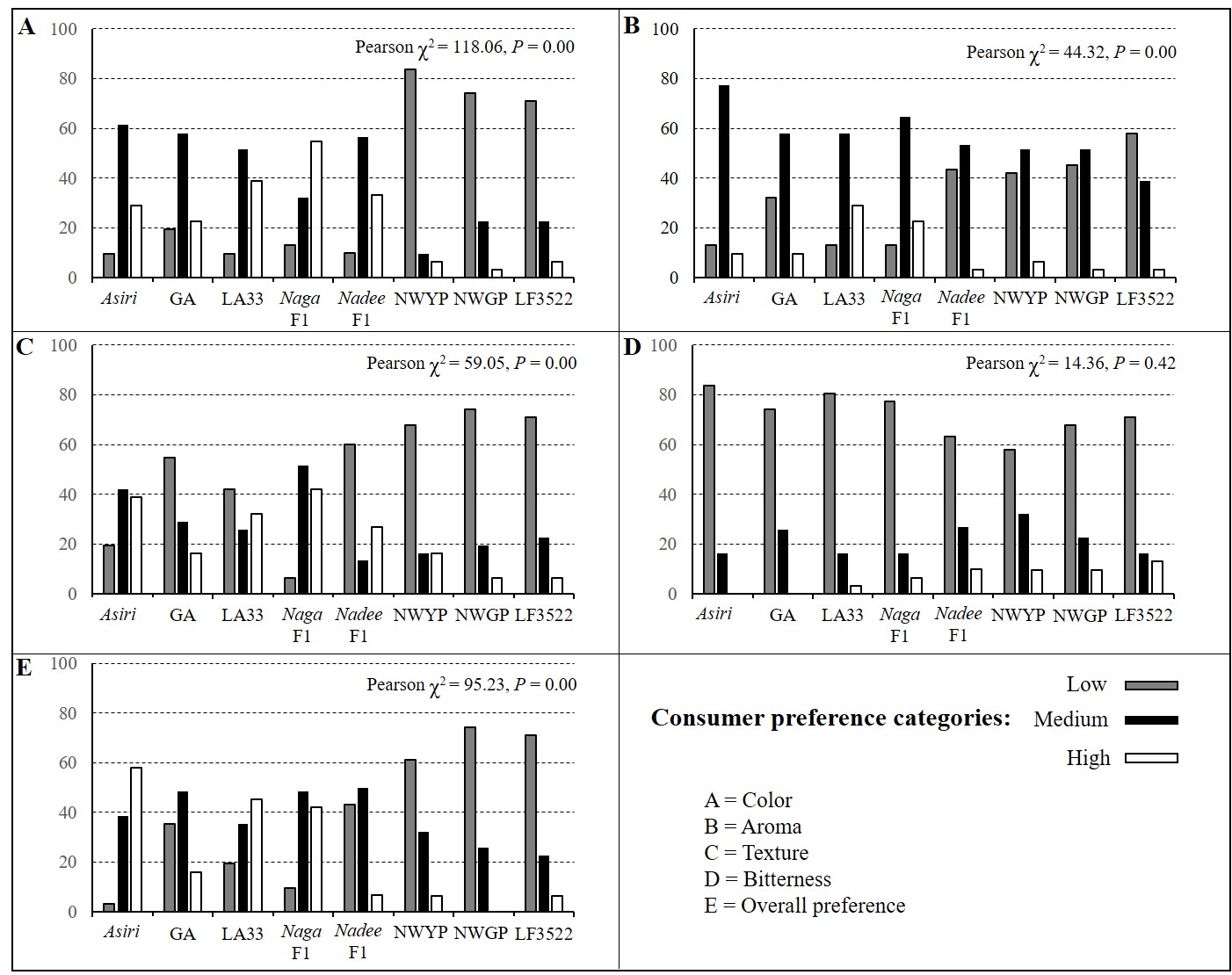

Supplement: S4 Fig — Y-axis represent the percentage panelists responded in the survey. Pearson ᵪ2 and P value are indicated in the top right corner. The color of the bar with respect to the preferred level is given according to the key given bottom-right. (JPG) [file pone.0215176.s004.jpg]
