# Supplementary material for: The establishment of the species-delimits and varietal-identities of the cultivated germplasm of Luffa acutangula and Luffa aegyptiaca in Sri Lanka using morphometric, organoleptic and phylogenetic approaches
Source: PLoS One. 2019 Apr 9;14(4):e0215176. doi: 10.1371/journal.pone.0215176 (PMC6456250; doi:10.1371/journal.pone.0215176)
Supplement: S1 Table — (DOCX) [file pone.0215176.s005.docx]

| **Abbreviation** | **Long term definition** |
| --- | --- |
| DHV | Number of Days to Harvest the first Vegetable since the flowering stage |
| DMF | Number of Days to the first Male Flower |
| FFN | First Flowering Node |
| FG | Fruit Girth |
| FL | Fruit Length |
| FN | Number of Fruits |
| FT | Flesh Thickness |
| FW | Fruit Width |
| GA | *Gannoruwa Ari* |
| HSW | Hundred Seed Weight |
| INL | Internode Length |
| INW | Internode Width |
| IPGR | International Plant Genetic Resources |
| IPM | Integrated Pest Management |
| LL | Leaf Length |
| LW | Leaf Width |
| MFR | Male to Female Ratio |
| NDH | Number of Days to Harvesting since the establishment of plants |
| NFF | Number of days to the first Female Flower |
| NLS | Number of Lateral Shoots |
| NP | Number of seeds per Pod |
| NWGP | *Niyan Watakolu* Green Peel |
| NWYP | *Niyan Watakolu* Yellow Peel |
| PDL | Peduncle Length |
| PL | Petiole Length |
| RDW | Root Dry Weight |
| RN | Number of Ribs |
| RTL | Root Length |
| SET | Seed Thickness |
| SL | Seed Length |
| ST | Skin Thickness |
| SW | Seed Width |
| TW | Total Weight |
| VGF | Vine Girth at the Flowering stage |
| VLF | Vine Length at the Flowering stage |

S1 Table
