# Supplementary material for: The establishment of the species-delimits and varietal-identities of the cultivated germplasm of Luffa acutangula and Luffa aegyptiaca in Sri Lanka using morphometric, organoleptic and phylogenetic approaches
Source: PLoS One. 2019 Apr 9;14(4):e0215176. doi: 10.1371/journal.pone.0215176 (PMC6456250; doi:10.1371/journal.pone.0215176)
Supplement: S2 Table — (DOCX) [file pone.0215176.s006.docx]

**S2 Table** Plant DNA barcoding markers and the PCR conditions employed in the analysis of DNA length polymorphisms

| DNA marker | Sequence (5’🡪3’) | PCR Profile | | | | | | | | | | Reference |
| --- | --- | --- | --- | --- | --- | --- | --- | --- | --- | --- | --- | --- |
|  |  | Initial denaturation | | Denaturation | | Primer  annealing | | Initial  extension | | Final  extension | |  |
|  |  | T (ºC) | Time | T (ºC) | Time | T (ºC) | Time | T (ºC) | Time | T (ºC) | Time |  |
| *trnH-psbA* | F- CGCGCATGGTGGATTCACAATCC | 98 | 45 sec | 98 | 10 sec | 64 | 30 sec | 72 | 40 sec | 72 | 10 min | Tate and Simpson (2003) |
|  | R- GTTATGCATGAACGTAATGCTC |  |  |  |  |  |  |  |  |  |  | Sang et al. (1997) |
| *rbcL* | F-ATGTCACCACAAACAGAGACTAAAGC | 98 | 45 sec | 98 | 10 sec | 55 | 30 sec | 72 | 40 sec | 72 | 10 mins | Levin et al. (2003) |
|  | R- GTAAAATCAAGTCCACCRCG |  |  |  |  |  |  |  |  |  |  | Kress and Erickson (2007) |
| *trnL-trnF spacer* | F- CGA AAT CGG TAG ACG CTA CG | 80 | 5 mins | 94 | 1 min | 50 | 1 min | 72 | 2 mins | 94 | 4 mins | Taberlet et al. (1991) |
|  | R- ATT TGA ACT GGT GAC ACG AG |  |  |  |  |  |  |  |  |  |  |  |
| *trnS^GCU^-trnG^UUC^* | F- AGA TAG GGA TTC GAA CCC TCG GT | 80 | 5 mins | 95 | 1 min | 66 | 4 min | 66 | 4 mins | 66 | 10 mins | Shaw et al. (2005) |
|  | R- GTA GCG GGA ATC GAA CCC GCA TC |  |  |  |  |  |  |  |  |  |  |  |
| *atpB-rbcL spacer* | F-GAAGTAGTAGGATTGATTCTC | 94 | 4 mins | 94 | 30 sec | 45 | 30 sec | 72 | 2 mins | 72 | 5 mins | Hoot and Taylor (2001) |
|  | R-TACAGTTGTCCATGTACCAG |  |  |  |  |  |  |  |  |  |  |  |
| *atpB gene* | F-TATGAGAATCAATCCTACTACTTCT | 92 | 3 mins | 92 | 1 min | 55 | 1 min | 72 | 3 mins | 72 | 7 mins | Hoot et al. (1995) |
|  | R-TCAGTACACAAAGATTTAAGGTCAT |  |  |  |  |  |  |  |  |  |  |  |
| *matK-trnT spacer* | F-GCATAAATATAYTCCYGAAARATAAGTGG | 95 | 1.5 mins | 95 | 30 sec | 48 | 1 min | 68 | 2 mins | 68 | 20 mins | Wicke and Quandt (2009) |
|  | R-TGGGTTGCTAACTCAATGG |  |  |  |  |  |  |  |  |  |  |  |
| *trnL (tRNA-leu* gene) | F-CGAAATCGGTAGACGCTACG  R-GGGGATAGAGGGACTTGAAC | 95 | 10 mins | 95 | 30 sec | 50 | 30 sec | 72 | 2 mins | 94 | 4 mins | Taberlet et al. (2006) |
| *ITS1-4* | F-TCC GTA GGT GAA CCT TGC GG  R-TCC TCC GCT TAT TGA TAT GC | 95 | 3 mins | 95 | 1 min | 55 | 1 min | 72 | 1.5 mins | 72 | 4 mins | White et al. (1990) |
