# Supplementary material for: The establishment of the species-delimits and varietal-identities of the cultivated germplasm of Luffa acutangula and Luffa aegyptiaca in Sri Lanka using morphometric, organoleptic and phylogenetic approaches
Source: PLoS One. 2019 Apr 9;14(4):e0215176. doi: 10.1371/journal.pone.0215176 (PMC6456250; doi:10.1371/journal.pone.0215176)
Supplement: S3 Table — (DOCX) [file pone.0215176.s007.docx]

S3 Table

| **Species** | **Variety** | **Voucher No.** | **Geographic origin** | **GenBank accessions** | | |
| --- | --- | --- | --- | --- | --- | --- |
|  |  |  |  | ***trnH-psbA*** | ***rbcL*** | ***ITS*** |
| *L. acutangula* | *Asiri* | DMB35 | Sri Lanka, Gannoruwa | MK279335 | MK279343 | MK271063 |
| *L. acutangula* | GA | DMB36 | Sri Lanka, Gannoruwa | MK279336 | MK279344 | MK271064 |
| *L. acutangula* | LA33 | DMB37 | Sri Lanka, Gannoruwa | MK279337 | MK279345 | MK271065 |
| *L. acutangula* | *Naga-F1* | DMB38 | Sri Lanka, Rajagiriya | MK279338 | MK279346 | MK271066 |
| *L. acutangula* | *Nadee-F1* | DMB39 | Sri Lanka, Rajagiriya | MK279339 | MK279347 | MK271067 |
| *L. aegyptiaca* | NWYP | DMB40 | Sri Lanka, Gannoruwa | MK279340 | MK279348 | MK271068 |
| *L. aegyptiaca* | NWGP | DMB41 | Sri Lanka, Wanathawilluwa | MK279341 | MK279349 | MK271069 |
| *L. aegyptiaca* | LF3522 | DMB42 | Sri Lanka, Thalakiriyagama | MK279342 | MK279350 | MK271070 |
| *L. acutangula* |  | 37 | India, Rajasthan | KF487491 |  | KF487333 |
| *L. acutangula* |  | 1548 | India, Rajasthan | KF487490 |  | KF487332 |
| *L. acutangula* |  | 1549 | Yemen | KF487492 |  | KF487335 |
| *L. aegyptiaca* |  | 5443 | Australia, Northern  Territory | KF487500 |  | KF487344 |
| *L. aegyptiaca* |  | 6308 | Australia,  Queensland | KF487503 |  | KF487347 |
| *L. aegyptiaca* |  | 300 | Indonesia, Lesser  Sunda Islands,  Flores | KF487501 |  | KF487345 |
| *L. aegyptiaca* |  | 4298 | Papua  New Guinea,  Milne Bay | KF487495 |  | KF487339 |
| *L. aegyptiaca* |  | 4117-5 | Samoa, Upolu | KF487504 |  | KF487348 |
| *L. aegyptiaca* |  | 3418 | Laos, Ban Thong | KF487496 |  | KF487340 |
| *L. aegyptiaca* |  | 3435 | Laos, Ban Thong | KF487497 |  | KF487341 |
| *L. aegyptiaca* |  | 22 | India, Tamil Nadu | KF487494 |  | KF487338 |
| *L. aegyptiaca* |  | 801 | Vietnam, Dalat | KF487502 |  | KF487346 |
| *L. aegyptiaca* |  | 2841 | Cultivated from  commercial  seeds | KF487499 |  | KF487343 |
| *L. aegyptiaca* |  | 2756 | Australia | KF487498 |  | KF487342 |
| *L. aegyptiaca* |  | 1550 | China, Yunnan | KF487493 |  | KF487337 |
| *L. astorii* |  | 1534 | Galapagos,  Santa Maria | KF487474 |  | KF487314 |
| *L. astorii* |  | 2164 | Galapagos,  Santa Maria | KF487481 |  | KF487321 |
| *L. astorii* |  | 15346 | Ecuador, Guayas | KF487470 |  | KF487311 |
| *L. astorii* |  | 5174 | Peru, Ancash | KF487471 |  | KF487312 |
| *L. astorii* |  | 8521 | Peru,  Lambayeque | KF487480 |  | KF487320 |
| *L. astorii* |  | 14901 | Peru, Ascope | KF487479 |  | KF487319 |
| *L. astorii* |  | 1182 | Peru,  Jequetepeque | KF487472 |  | KF487313 |
| *L. astorii* |  | 2500 | Ecuador, Guayas | KF487476 |  | KF487316 |
| *L. astorii* |  | 1547 | Ecuador, Guayas | KF487475 |  | KF487315 |
| *L. astorii* |  | 63536 | Ecuador, Guayas  (Isla Puna) | KF487477 |  | KF487318 |
| *L. astorii* |  | 63011 | Ecuador, Guayas  (Isla Puna) | KF487478 |  | KF487317 |
| *L. echinata* |  | 13381 | India,  Uttar Pradesh | KF487507 |  | KF487351 |
| *L. echinata* |  | 1481 | India | KF487505 |  | KF487349 |
| *L. echinata* |  | 123 | Sudan, Khartoum | KF487506 |  | KF487350 |
| *L. graveolens* |  | 2758 | India, USDA | KF487509 |  | KF487353 |
| *L. graveolens* |  | 101401 | India,  Uttar Pradesh | KF487508 |  | KF487352 |
| *L. operculata* |  | 1183 | Panama, Cocle | KF487483 |  | KF487323 |
| *L. operculata* |  | 1545 | Brazil, Sa˜o Paulo | KF487484 |  | KF487324 |
| *L. operculata* |  | 19937 | Peru, Loreto | KF487482 |  | KF487322 |
| *L. quinquefida* |  | 4742 | Mexico, Sonora | KF487485 |  | KF487326 |
| *L. quinquefida* |  | 1546 | Mexico, Sinaloa | KF487486 |  | KF487325 |
| *L. quinquefida* |  | 1440 | USA, Louisiana | KF487487 |  | HQ201986 |
| *L. quinquefida* |  | 13251 | Nicaragua,  Dep. Leon  (Isla  Momotombito) | KF487488 |  | KF487328 |
| *L. quinquefida* |  | 274 | Mexico, Sonora | KF487489 |  | KF487329 |
| *L. saccata* |  | 1482 | Australia | KF487510 |  | KF487354 |
| *L. saccata* |  | 3352 | Australia | KF487511 |  | KF487355 |
| *L. saccata* |  | 4499 | Australia,  Kimberley | KF487512 |  | KF487356 |
