# Supplementary material for: The establishment of the species-delimits and varietal-identities of the cultivated germplasm of Luffa acutangula and Luffa aegyptiaca in Sri Lanka using morphometric, organoleptic and phylogenetic approaches
Source: PLoS One. 2019 Apr 9;14(4):e0215176. doi: 10.1371/journal.pone.0215176 (PMC6456250; doi:10.1371/journal.pone.0215176)
Supplement: S4 Table — (DOCX) [file pone.0215176.s008.docx]

S4 table

| Principle Component | PC1 | PC2 | PC3 | PC4 | PC5 |
| --- | --- | --- | --- | --- | --- |
| Eigenvalue | 12.885 | 4.73 | 3.706 | 1.668 | 1.299 |
| Covariance | 0.403 | 0.148 | 0.116 | 0.052 | 0.041 |
