# Supplementary material for: The establishment of the species-delimits and varietal-identities of the cultivated germplasm of Luffa acutangula and Luffa aegyptiaca in Sri Lanka using morphometric, organoleptic and phylogenetic approaches
Source: PLoS One. 2019 Apr 9;14(4):e0215176. doi: 10.1371/journal.pone.0215176 (PMC6456250; doi:10.1371/journal.pone.0215176)
Supplement: S5 Table — (DOCX) [file pone.0215176.s009.docx]

S5 table

| Principle Component | PC1 | PC2 | PC3 | PC4 | PC5 |
| --- | --- | --- | --- | --- | --- |
| Eigenvalue | 4.2637 | 0.3639 | 0.223 | 0.1302 | 0.0191 |
| Covariance | 0.853 | 0.073 | 0.045 | 0.026 | 0.004 |
